# Antileukemic potential of methylated indolequinone MAC681 through immunogenic necroptosis and PARP1 degradation

**DOI:** 10.1186/s40364-024-00594-w

**Published:** 2024-05-04

**Authors:** Barbora Orlikova-Boyer, Anne Lorant, Sruthi Reddy Gajulapalli, Claudia Cerella, Michael Schnekenburger, Jin-Young Lee, Ji Yeon Paik, Yejin Lee, David Siegel, David Ross, Byung Woo Han, Thi Kim Yen Nguyen, Christo Christov, Hyoung Jin Kang, Mario Dicato, Marc Diederich

**Affiliations:** 1 Laboratoire de Biologie Moléculaire du Cancer, BAM3 Pavillon 2, 6A Rue Nicolas-Ernest Barblé, L-1210 Luxembourg, Luxembourg; 2 Department of Pharmacy, Research Institute of Pharmaceutical Sciences & Natural Products Research Institute, College of Pharmacy, Seoul National University, 1, Gwanak-Ro, Gwanak-Gu, Seoul, 08826 Republic of Korea; 3 Skaggs School of Pharmacy and Pharmaceutical Sciences, University of Colorado Anschutz Medical Campus, Aurora, CO 80045 USA; 4 Faculté de Médecine, Université de Lorraine, Nancy, France; 5 Department of Pediatrics, Seoul National University College of Medicine, Seoul National University Cancer Research Institute, Seoul National University Children’s Hospital, Seoul, 03080 Republic of Korea; 6 Present address: Department of Biological Sciences, Keimyung University, Daegu, 42601 Republic of Korea

**Keywords:** Indolequinone, NAD, PARP1, OXPHOS, Chronic myeloid leukemia, Necrosis, Immunogenic cell death, 3-aminobenzamide

## Abstract

**Background:**

Despite advancements in chronic myeloid leukemia (CML) therapy with tyrosine kinase inhibitors (TKIs), resistance and intolerance remain significant challenges. Leukemia stem cells (LSCs) and TKI-resistant cells rely on altered mitochondrial metabolism and oxidative phosphorylation. Targeting rewired energy metabolism and inducing non-apoptotic cell death, along with the release of damage-associated molecular patterns (DAMPs), can enhance therapeutic strategies and immunogenic therapies against CML and prevent the emergence of TKI-resistant cells and LSC persistence.

**Methods:**

Transcriptomic analysis was conducted using datasets of CML patients' stem cells and healthy cells. DNA damage was evaluated by fluorescent microscopy and flow cytometry. Cell death was assessed by trypan blue exclusion test, fluorescent microscopy, flow cytometry, colony formation assay, and in vivo Zebrafish xenografts. Energy metabolism was determined by measuring NAD^+^ and NADH levels, ATP production rate by Seahorse analyzer, and intracellular ATP content. Mitochondrial fitness was estimated by measurements of mitochondrial membrane potential, ROS, and calcium accumulation by flow cytometry, and morphology was visualized by TEM. Bioinformatic analysis, real-time qPCR, western blotting, chemical reaction prediction, and molecular docking were utilized to identify the drug target. The immunogenic potential was assessed by high mobility group box (HMGB)1 ELISA assay, luciferase-based extracellular ATP assay, ectopic calreticulin expression by flow cytometry, and validated by phagocytosis assay, and in vivo vaccination assay using syngeneic C57BL/6 mice.

**Results:**

Transcriptomic analysis identified metabolic alterations and DNA repair deficiency signatures in CML patients. CML patients exhibited enrichment in immune system, DNA repair, and metabolic pathways. The gene signature associated with BRCA mutated tumors was enriched in CML datasets, suggesting a deficiency in double-strand break repair pathways. Additionally, poly(ADP-ribose) polymerase (PARP)1 was significantly upregulated in CML patients’ stem cells compared to healthy counterparts. Consistent with the CML patient DNA repair signature, treatment with the methylated indolequinone MAC681 induced DNA damage, mitochondrial dysfunction, calcium homeostasis disruption, metabolic catastrophe, and necroptotic-like cell death. In parallel, MAC681 led to PARP1 degradation that was prevented by 3-aminobenzamide. MAC681-treated myeloid leukemia cells released DAMPs and demonstrated the potential to generate an immunogenic vaccine in C57BL/6 mice. MAC681 and asciminib exhibited synergistic effects in killing both imatinib-sensitive and -resistant CML, opening new therapeutic opportunities.

**Conclusions:**

Overall, increasing the tumor mutational burden by PARP1 degradation and mitochondrial deregulation makes CML suitable for immunotherapy.

**Supplementary Information:**

The online version contains supplementary material available at 10.1186/s40364-024-00594-w.

## Introduction

Tyrosine kinase inhibitors (TKIs) represent the frontline treatment against chronic myeloid leukemia (CML), characterized by the t(9:22) chromosomal translocation, resulting in the breakpoint cluster region-Abelson (BCR-ABL)1 fusion oncogene with constitutively activated Abl tyrosine kinase activity. Despite TKI efficacy, resistant mutants turn CML into a controllable yet persisting chronic disease, interfering with the patient’s quality of life [1–3]. Moreover, one-third of newly diagnosed patients develop primary or secondary resistance toward approved TKIs, and clinical relapse occurs [4]. Imatinib resistance originates from BCR-ABL-dependent and -independent mechanisms, including leukemia stem cell (LSC) treatment insensitivity. LSCs and TKI/therapy-resistant cells depend on mitochondrial metabolism and oxidative phosphorylation (OXPHOS) to meet their energy demands [5, 6], in contrast to mature CML cells or normal hematopoietic stem cells (HSCs).

The goal of therapeutic approaches for CML treatment is to eliminate LSCs by identifying and targeting genetic alterations and molecular pathways that contribute to their survival and resistance to apoptosis. Efforts must be undertaken to develop combination therapies targeting tyrosine kinases with novel drug candidates that specifically target genes or molecules necessary for the survival mechanisms of CML LSCs [7]. Accordingly, identifying and targeting rewired energy metabolism is a relevant strategy for developing non-ABL-related therapeutic options for treating TKI-resistant CML patients, preventing the appearance of TKI-resistant cells, and LSC persistence.

Nicotinamide adenine dinucleotide (NAD) is a substrate for NAD^+^-dependent enzymes and a major coenzyme in bioenergetic processes. The NAD^+^/NADH ratio modulates metabolic processes, including oxidative phosphorylation (OXPHOS), tricarboxylic acid (TCA) cycle, fatty acid oxidation (FAO), and glycolysis. NADH serves as a central hydride donor to mitochondrial ATP synthesis. Maintaining NAD^+^ levels and the NAD^+^/NADH ratio is crucial for mitochondrial function, ATP production, and homeostasis. Hence, NAD metabolism is a master regulator linking metabolic processes to OXPHOS [8]. Most cells replenish their NAD^+^ pool primarily via the NAD^+^ salvage pathway by recycling nicotinamide (NAM), generated by NAD^+^-consuming enzymes predominantly by poly (ADP-ribose) polymerase 1 (PARP1) and sirtuin (SIRT)1 [9, 10].

Moreover, PARP1 is a critical regulator of DNA damage repair. BCR-ABL-expressing CML cells accumulate specific defects in DNA repair pathways, including the downregulation of breast cancer gene (BRCA)1 and the DNA-dependent protein kinase (DNA-PK) catalytic subunit. Both are critical components of homologous recombination (HR) and nonhomologous DNA end joining (cNHEJ), respectively [11, 12]. Hence, CML cells must rely on a highly error-prone PARP1-dependent alternative (ALT)-NHEJ machinery to repair DSBs [13], likely contributing to disease progression by causing genomic instability. As a result, CML cells refractory to TKIs are genetically unstable, which may cause relapse and malignant progression to advanced and terminal disease states [14]. These BCR-ABL-specific deficiencies present an opportunity to exploit synthetic lethality by targeting PARP1.

The naturally occurring antitumor indolequinone mitomycin C is known to alkylate DNA upon enzymatic bioreductive activation [15]. Moreover, an extensive library of indolequinone derivatives, differing in the substituent on the 2-position of the indole ring, was tested against pancreatic cancer with promising results [16, 17] and thus provided proof of the anticancer potential of this family of compounds. Qin et al. underline the role of indole alkaloids in targeting different forms of regulated cell death. Accordingly, the investigation and semisynthesis of indole alkaloids to target various cancer types are believed to be advantageous in creating new molecules with improved biological properties, which can aid in developing novel approaches to cancer treatment [18].

In addition to the cytotoxic activity, whether by apoptosis or controlled necrosis, the activation of the immune-mediated destruction of tumor cells is deeply involved in the curative effect of a given anticancer agent. There is a growing interest in promoting anti-tumor immune responses as a potential alternative strategy for cancer treatment [19]. Accordingly, the induction of immunogenic cell death (ICD) is an attractive approach to fighting cancer malignancies [20]. Pharmacological mediators of ICD stimulate the host’s immune system via the release of damage‐associated molecular patterns (DAMPs), eventually triggering a persistent immune response [21].

We investigated a TKI-independent, immunogenic cell death mechanism triggered by a novel, methylated indolequinone (MAC681). This compound targets PARP1 degradation, exacerbating mitochondrial dysfunction and inducting a non-canonical, necroptotic-like cell death. We used MAC681-treated, dying, or dead cells to demonstrate immunogenic vaccination against myeloid leukemia.

## Materials and methods

### Computational analysis of public CML datasets

This study utilized several publicly available datasets, which were processed as follows:

A) GSE5550 [22]: 17 patients’ bone marrow samples sorted for CD34+ hematopoietic stem and progenitor cells, 8 healthy donors, and 9 untreated CML patients in chronic phase were downloaded from GEO. The samples were processed and normalized using the robust Multi-Array Average (RMA) expression measure from the “affy” package in R [23]. All the probes were analyzed separately; if multiple probes representing the same gene were present, only the most significant probe was kept.
B) GSE97562 [24] and GSE47927 [25]: A total of 19 patients’ bone marrow samples sorted for CD34+, 8 healthy donors, and 11 untreated CML patients in chronic phase raw CEL files were downloaded from GEO (Supp. Table S1A). Samples were processed and normalized using the RMA expression measure from the “affy” package in R [23]. To correct for the batch effect between datasets, we employed the combat function of the "sva" R package [26].
C) The Microarray Innovations in LEukemia (MILE) study, GSE13159 [27]: A total of 66 untreated CML patients’ bone marrow mononuclear cells raw CEL files were downloaded from GEO. Samples were processed and normalized using the robust RMA expression measure from the “affy” package in R [23]. Samples were divided into 4 groups based on the quartiles of PARP1 expression. The high and low quartiles were subsequently used for the differential expression analysis.
D) The Cancer Cell Line Encyclopedia (CCLE) dataset: Gene expression transcript per million (TPM) normalized reads from all the cancer cell lines (*n* = 1165) were downloaded from the Depmap portal website (https://depmap.org/portal/download/all/) [28].

#### Differential expression analysis

To identify differentially expressed genes (DEGs) between healthy and CML CD34^+^ cells or between CML patients with high and low expression of PARP1, we utilized the “limma” R package [29]. We applied a threshold of |log2(FC)|> 0.3 and a false discovery rate (FDR) < 0.05. The resulting DEGs were visualized using volcano and generated using the “ggplot2” R package [30] (https://ggplot2.tidyverse.org).

#### Pathways and enrichment analysis

We performed functional enrichment analysis using GO terms, GSE analysis (GSEA), and Reactome pathways to analyze the identified DEGs comprehensively. GO terms and GSEA analysis were performed using “fgsea” and “msigdb” R packages. Reactome enrichments were performed on the web-based application [31] (https://reactome.org/). Pathways were considered significant when the FDR ≤ 0.05. We re-grouped each pathway for its higher hierarchical annotation in Reactome and calculated a weighted percentage of pathways enriched. Genes belonging to the NAD^+^ metabolome pathway were collected from Xie et al. [10]. Enrichment was tested with a Fisher exact test.

### Compounds

6-Methoxy-1,2-dimethyl-3-(2,4,6-trifluoropheoxy)methylindole-4,7-dione (MAC681) was a generous gift from Prof. Christopher J Moody, School of Chemistry, University of Nottingham, UK. The compound has a molecular weight of 365.3 g/mol and was solubilized in 100% dimethyl sulfoxide (DMSO) (Sigma-Aldrich, Bornem, Belgium) at a stock concentration of 5 mM. Aliquots were stored at -80°C. All pharmacological inhibitors used in this study are summarized in Supplementary Table 1.

### Cell culture

Chronic myeloid leukemia cell lines (K-562, MEG-01) and acute myeloid leukemia (AML) cell lines (U-937, HL-60, MOLM-13, MV4-11) were purchased from Deutsche Sammlung für Mikroorganismen und Zellkulturen (DSMZ; Braunschweig, Germany) and cultured in RPMI 1640 medium (Lonza, Basel, Switzerland) supplemented with 10% (v/v) fetal calf serum (FCS; Biowest, Nuaille, France) and 1% (v/v) antibiotic–antimycotic (Lonza, Basel, Switzerland). Chronic myeloid leukemia KBM5 cells were kindly donated by Dr. Bharat B. Aggarwal and were cultured in IMDM media supplemented with 10% (v/v) FCS and 1% (v/v) antibiotic–antimycotic. J774A1 cells were purchased from the Seoul National University cell bank and cultured in DMEM/high glucose media (Hyclone, GE Healthcare Life Sciences, Utah, USA) supplemented with 10% (v/v) FCS and 1% (v/v) antibiotic–antimycotic. Acute myeloid leukemia OCI-AML3 cells were purchased from DSMZ and cultured in Alpha MEM medium (Lonza, Basel, Switzerland) supplemented with 15% (v/v) FCS and 1% (v/v) antibiotic–antimycotic. C1498 cells (ATCC® TIB-49TM) were maintained according to the American type culture collection (ATCC) protocol with high glucose Dulbecco's Modified Eagle Media (Gibco, Catalog No 11995–065) with 10% fetal bovine serum (Biowest, Riverside, MO, USA) and 1% (v/v) of an antibiotics-antimycotics solution (Lonza, Walkersville, MD, USA). Passages of less than 10 were used for the vaccination experiments.

After ethical approval, peripheral blood mononuclear cells (PBMCs) were obtained from healthy adult human volunteers (Red Cross, Luxembourg, Luxembourg). PBMCs from healthy donors and CML patients were isolated by Ficoll™ (GE Healthcare, Roosendaal, The Netherlands) density gradient centrifugation from freshly collected buffy coats (400 g, 20 min). After isolation, PBMCs from healthy donors were washed, counted, and resuspended at a cell concentration of 2.0 × 10^6^ cells/mL in RPMI 1640 supplemented with 10% (v/v) FCS and 1% (v/v) antibiotic–antimycotic and kept overnight in a humidified atmosphere in the incubator at 37°C and 5% CO_2_ before the treatment. Primary CML cells were cultured in RPMI 1640 supplemented with 10% heat-inactivated fetal bovine serum.

Primary CD34^+^ progenitor/stem cells were isolated from human umbilical cord blood obtained from the Clinique Bohler (Robert Schuman Hospital, Luxembourg, Luxembourg) with the written informed consent of parents with the approval of the National Research Ethics Committee of Luxembourg. First, mononucleated cells were isolated by Ficoll™ (GE Healthcare, Roosendaal, The Netherlands) density gradient centrifugation. CD34^+^ cells were then purified from mononucleated cells using magnetic cell sorting following the manufacturer's instructions (MACS Miltenyi, Utrecht, The Netherlands). High purity CD34^+^ cells were treated after 3 days of amplification in Stemline® II Hematopoietic Stem cell Expansion Medium (Sigma-Aldrich) supplemented with 1% antibiotic–antimycotic (BioWhittaker®, Lonza, Verviers, Belgium), 4 mM L-glutamine (BioWhittaker®), 50 ng/mL stem cell factor (ReliaTech, Wolfenbüttel, Germany), and 10 ng/mL interleukin (IL)-3 (ReliaTech, Wolfenbüttel, Germany).

RPMI 1788 peripheral blood B lymphocytes were purchased from ATCC and cultured in RPMI 1640 medium (Lonza, Basel, Switzerland) supplemented with 20% (v/v) FCS and 1% (v/v) antibiotic–antimycotic. All cell lines were cultured at 37°C and 5% CO_2_ in a humidified atmosphere and were regularly tested for mycoplasma infection (Mycoalert™, Lonza USA) per the manufacturer’s instructions.

### Cell proliferation and viability

Cell number and viability were determined using a semi-automated image-based Cedex XS cell counter (Innovatis AG, Roche, Basel, Switzerland) and by Malassez counting chamber using the Trypan Blue exclusion assay (Lonza, Basel, Switzerland). Moreover, to quantify metabolically active cells, intracellular ATP levels were measured by CellTiter-Glo® Luminescent Cell Viability Assay (Promega, Leiden, Netherlands) following the manufacturer's protocol and as previously described [32], and luminescence was measured using an Orion Microplate Luminometer (Berthold, Pforzheim, Germany).

To calculate differential toxicity, the viability of healthy cells was compared to the viability of cancer cells (normal / cancer cells) at specific concentrations and time points. The difference in viability was expressed in terms of fold change.

### Protein extraction and western blots

Cells were washed with cold 1 × phosphate buffer saline (PBS). For whole cell extracts, pellets were lysed using M-PER Mammalian Protein Extraction Reagent (Pierce, Erembodegem, Belgium) completed with a protease inhibitor cocktail (Complete; Roche, Luxembourg, Belgium), 1 µM phenylmethylsulfonyl fluoride, 1 mM sodium orthovanadate, 5 mM sodium fluoride and incubated 15 min at 4°C on a shaking platform; then centrifuged 15 min at 15 000 g and 4°C for clarification. For cytoplasmic and nuclear cell extracts, pellets were lysed using buffer A (Supplementary Table 2) for 15 min at 4°C, followed by adding of IGEPAL 10% (MP Biomedicals, LLC, Illkirch, France) and centrifuged for 1 min at 13000 rpm and 4°C. After collection of cytoplasmic extracts, lysis buffer C (Supplementary Table 2) was added to the nuclear pellets and vortexed for 20 min at 4°C, followed by 5 min centrifugation at 10000 rpm at 4°C, and nuclear pellets were collected. Twenty μg of proteins were mixed with 2 × Laemmli buffer and boiled. Samples were loaded onto a sodium dodecyl polyacrylamide gel (stacking: 4%, resolving: 10%). After migration, proteins were transferred to a polyvinylidene difluoride membrane (GE Healthcare, Roosendaal, The Netherlands), followed by blocking in 1 × PBS 0.1% Tween® 20 (Sigma-Aldrich) containing 5% milk or bovine serum albumin. Membranes were incubated with selected primary antibodies (Supplementary Table 3). After incubation, membranes were washed with 1 × PBS 0.1% Tween and incubated with the appropriate secondary antibodies (Santa Cruz Biotechnology or BD Biosciences) for 1 h at room temperature (RT). After washing, the membranes were revealed using the ECL Plus Western Blotting Detection System Kit (GE Healthcare). Chemiluminescence was detected with an Imagequant LAS Mini (GE Healthcare). The bands were quantified using ImageJ (from the National Institutes of Health, Bethesda, MD, USA; http://rsbweb.nih.gov/ij/). Sample loading was controlled using β-actin.

### Quantification of apoptosis and necrosis

The percentage of apoptotic cells was quantified as the fraction of cells showing fragmented nuclei, as assessed by fluorescence microscopy using an IX81(MT10) Olympus microscope (Olympus, Aartselaar, Belgium) after staining with Hoechst 33342 (Sigma-Aldrich). For the quantification of necrosis/secondary necrosis, a propidium iodide (PI) staining was performed in addition to the Hoechst 33342 staining, and the number of PI-positive cells was counted similarly to apoptotic cells. Cell death was also confirmed by PI staining analyzed by flow cytometry (FACScalibur, BD Biosciences, San José, CA, USA) according to the manufacturer protocol (BD Biosciences). Data were recorded statistically (10000 events/sample) using the CellQuest software (BD Biosciences) and analyzed using Flow-Jo 10.8.1 software (Tree Star, Inc., Ashland, OR, USA).

### Analysis of mitochondrial membrane potential

The loss of mitochondrial membrane potential was analyzed by MitoTracker Red staining (Molecular Probes/Invitrogen, Merelbeke, Belgium). Briefly, 1 × 10^6^ cells were incubated for 20 min at 37°C with 100 nM MitoTracker Red. Fluorescence intensity was measured by flow cytometry (FACScalibur, BD Biosciences, San José, CA, USA). Mitochondrial oxidative phosphorylation uncoupler carbonyl cyanide m-chlorophenyl hydrazone (CCCP; 50 µM, 20 min) was used as a positive control. Cell death induction was parallelly analyzed by flow cytometry by PI uptake after 15 min staining with PI (0.5 µg/mL). DMSO (50%, 1 min) was used as a positive control. Events were recorded (10000 events/sample) using the CellQuest software. Data were further analyzed with the FlowJo 10.8.1 software.

### mRNA expression analysis

According to the manufacturer’s instructions, total RNA was extracted using NucleoSpin RNA II columns (Macherey–Nagel, Hœrdt, France). cDNA was synthesized by reverse transcription of 1 μg total RNA with M-MLV Reverse Transcriptase, RNase H Minus, and Point Mutant reverse transcriptase (Promega, Leiden, The Netherlands) using oligo(dT) primers (Promega). cDNAs were used as a template for subsequent quantification by real-time PCR in a reaction mixture containing 1 × Power SYBR® Green PCR Master Mix (Applied Biosystems, Halle, Belgium) and 0.1 μM of each primer (sequences available on request). Amplification was performed on an ABI 7300 real-time PCR system (Applied Biosystems, Lennik, Belgium). The amount of target gene was normalized to the endogenous level of β-actin using the 2^−ΔCT^ method.

### Indirect immunofluorescence

After treatment with MAC681, K-562 cells were fixed and permeabilized according to the manufacturer's instructions using the BD Cytofix/Cytoperm Kit (Becton Dickinson, Erembodegem, Belgium). Incubations with primary antibodies were performed in BD Perm/Wash solution for 1 h at RT with one of the following antibodies: anti-phospho histone H2A.X (Cell Signaling, 9718S) 1:80 dilution, apoptosis-inducing factor (AIF; Santa Cruz Biotechnology, sc-13116) 1:100 dilution. After washing twice with 1 × PBS, cells were incubated with the corresponding secondary antibody at 1:50 dilution at RT for 30 min on a shaking platform (mouse, Alexafluor 488, green fluorescence; rabbit or mouse, Alexafluor 568, red fluorescence; Invitrogen/Molecular Probes, Merelbeke, Belgium). After two additional washing steps with 1 × PBS, cells were analyzed by flow cytometry (FACScalibur, BD Biosciences, San José, CA, USA) and afterward counterstained with Hoechst 33342 (DNA specific, blue fluorescence) and monitored by fluorescence microscopy (Olympus, Aartselaar, Belgium). The images were analyzed using the Cell^M software (Olympus Soft Images Solutions GMBH, Germany).

### Human CXCL8/IL-8 immunoassay

IL-8 concentrations in culture supernatants of activated K-562 cells were measured by sandwich ELISA (R&D Systems, Abingdon, United Kingdom). According to the manufacturer's guide, 50 μL of cell supernatants were added with 100 μL of Assay Diluent to anti-IL-8 pre-coated wells, followed by 2 h incubation at RT. After washing, a polyclonal peroxidase-conjugated anti-IL-8 antibody was added for another 1 h at RT. Colorimetric visualization and protein dosage were developed by adding the H_2_O_2_ + tetramethylbenzidine-containing substrate. After a 30 min reaction at RT in the dark, the enzymatic reaction was stopped by the addition of H_2_SO_4,_ and optical densities were measured at a wavelength of 450 nm with a reference wavelength of 540 nm (Molecular Devices, LLC, San Jose, CA, USA).

### Colony formation assay (CFA)

For colony formation assays, 10^3^ cells/mL treated with MAC681 at indicated concentrations for 8 h were grown in a semi-solid methylcellulose medium (MethoCult™ H4230, StemCell Technologies Inc., Vancouver, Canada) supplemented with 10% FBS. Colonies were detected after 10 days of culture by adding 1 mg/mL 3-(4,5-dimethylthiazol-2-yl)-2,5-diphenyltetrazolium bromide (MTT) reagent (Sigma-Aldrich) and were scored by Image J 1.51 software (US National Institute of Health, Bethesda, MD, USA) [33].

### Transmission electron microscopy (TEM)

Cells were pelleted and fixed for 4 h in 2.5% glutaraldehyde (Euromedex, Mundolsheim, France) in 0.1 M sodium cacodylate buffer, pH 7.2 (Euromedex). Cells were then rinsed with sodium cacodylate buffer and postfixed in 1% osmium tetroxide (Euromedex) for 1 h at RT. Samples were washed and then dehydrated through a graded series of ethanol solutions to water followed by propylene oxide and then infiltrated in 1:1 propylene oxide/poly Bed 812 (Euromedex). Samples were kept overnight embedded in Poly Bed 812, mounted in molds, and left to polymerize in an oven at 56°C for 48 h. Ultrathin Sects. (70–90 nm) were obtained with a Reichert-Jung Ultracut S microtome (Wien, Austria). Sections were stained with uranyl acetate and lead citrate and examined with a CM12 transmission electron microscope (Philips, Eindhoven, The Netherlands).

### Measurement of extracellular ATP content

Extracellular ATP levels in the supernatant were assessed by the ENLITEN® ATP Assay System bioluminescence detection kit (Promega, Leiden, The Netherlands) following the manufacturer's protocol. Briefly, K-562 cells were seeded at 3 × 10^5^ cells/mL and treated with the indicated concentration of MAC681 for the indicated time. After the treatment, supernatants were collected by centrifugation, and 100 µL of reconstituted rluciferase/luciferin (rL/L) reagent was added to 100 µL of supernatant. The light output was measured immediately on an Orion Microplate Luminometer (Berthold, Pforzheim, Germany).

### Analysis of ectopic calreticulin expression

3 × 10^5^ cells /mL were cultured in 10 mL and treated at indicated concentrations with MAC681 for 10 h. Cells were collected, washed twice with 1 × PBS, and fixed in 0.25% paraformaldehyde in 1 × PBS for 5 min. After washing twice in cold 1 × PBS, cells were incubated for 40 min with the primary antibody for calreticulin (CRT; Abcam, 2907), diluted in cold blocking buffer (2% FBS in 1 × PBS) in 1:50 dilution, followed by washing and incubation with the Alexa fluor 488-conjugated monoclonal secondary antibody in a blocking buffer for 40 min. Each sample was then analyzed by FACS to detect cell surface CRT, and the median fluorescent intensity (MFI) was used to quantify the results.

### Detection of HMGB1 release

Cells were seeded at 3 × 10^5^ in 1 mL of medium. After indicated time points, cells were centrifuged, and the supernatant was collected and immediately stored at -80 ºC. Quantification of high mobility group box (HMGB) 1 release in the supernatants was assessed by enzyme-linked immunosorbent assay kit from IBL International (Tecan Benelux, Mechelen, Belgium) according to the manufacturer's instructions.

### Phagocytosis of MAC681-treated cells by J774A1 macrophages

K-562 cells were stained with CellTracker Red CMTPX dye (Thermofisher Scientific) for 1.5 h. After staining, cells were resuspended in a fresh RPMI medium, treated with 5 µM MAC681, and incubated for 16 h. J774A1 cells were stained with CellTracker Green CMFDA dye (Thermofisher Scientific) for 2 h and allowed to attach to the culture plate. MAC681-treated CML cells were co-cultured with J774A1 for 2 h with an effector-target ratio of 2:1. To confirm the level of phagocytosis, CML cells not interacting with J774A1 were eliminated by washing with 1 × PBS. The level of phagocytosis was quantified by counting the number of red K-562 cells phagocytized by green macrophages.

### Zebrafish xenografts

After mating, fertilized eggs were incubated in Danieau's solution with 0.003% of phenylthiourea (PTU) at 28.5°C for 48 h. Micropipettes for injection and anesthesia were generated from a 1.0 mm glass capillary (World Precision Instruments, FL, USA) using a micropipette puller (Shutter Instrument, USA). Forty-eight hours post-fertilization, zebrafish were anesthetized in 0.02% tricaine (Sigma-Aldrich, MO) and immobilized on an agar plate. Primary blasts were treated with indicated compounds for 8 h and 12 h. Then, cells were stained for an additional 2 h with 4 µM of cell tracker CM-Dil dye (Invitrogen). 100–200 primary blasts were injected into the yolk sac by microinjection (PV820 microinjector, World Precision Instruments, FL, USA). Subsequently, zebrafish were incubated in 24-well plates containing Danieau's solution with 0.003% phenylthiourea (PTU) at 28.5°C for 72 h. Fishes were then immobilized in a drop of 3% methylcellulose in Danieau's solution on a glass slide. Pictures were taken by fluorescence microscopy (Leica DE/DM 5000B). Fluorescent tumors were quantified by Image J software (US National Institute of Health, Bethesda, MD, USA).

### In vivo vaccination assay using a syngeneic mouse model

Mice were maintained according to the Institutional Animal Care and Use Committee (IACUC) standards followed by the Seoul National University, South Korea. This study was approved by Seoul National University IACUC (SNU-211222–5). All mice had unlimited water and food and were housed in pathogen-free cages containing wood shavings and bedding with a 12 h light/dark cycle and controlled room temperature of 25 °C. C57BL/6 J mice were purchased from Junga Bio Co., Ltd Company (Seoul, South Korea).

5 × 10^5^ C1498 cells were incubated with 5 µM MAC681 or 2.5 µM cytarabine and 0.5 µM doxorubicin for 24 h, washed once in 1 × PBS, and cell death was analyzed by trypan blue staining. These treated compound-free cells were injected subcutaneously with 100 µl PBS for vaccination into the right flanks of the 7-week-old C57BL/6 mice. Non-vaccination mice were given 100 µl PBS as the negative control group. The tumor incidence on the right flanks of the mice throughout the vaccination assay did not appear. One week later, all mice were challenged with 5 × 10^5^ C1498 untreated cells in the left flank of the mice. Tumor volumes were estimated using the formula = length x width x height mm^3^. Every other day, tumor growth was measured on the left flank with a digital Vernier caliper SD500-150PRO (Sincon, South Korea). Mice were sacrificed using 5% CO2 inhalation when tumor volumes reached > 600 mm^3^ in the non-vaccinated control group. All tumors were fixed in 4% paraformaldehyde for 24 h.

### Separate measurement of cellular NAD^+^ and NADH

Separate measurements of oxidized (NAD^+)^ and reduced (NADH) forms were assessed with the NAD/NADH-Glo™ Assay (Promega) following the manufacturer's protocol. Briefly, K-562 cells were seeded at 3 × 10^5^ cells/mL and treated with the indicated concentration of MAC681 for the indicated time. After the treatment, the concentration of cells was quantified by trypan blue exclusion assay, and cells were centrifuged and resuspended in 1 × PBS at a concentration of 5 × 10^5^/mL. The cells were lysed in the base solution with 1% dodecyltrimethylammonium bromide, then split into separate wells for acid (NAD^+^) and base (NADH) treatments. After 15 min heating at 60°C, the samples were incubated for 10 min at room temperature. 25 μl of Trizma® base (Sigma-Aldrich) was added to each well of acid-treated samples, and 50 μl of HCl/Trizma® solution was added to each well of base-treated samples. 50 μl of NAD/NADH-Glo™ Detection Reagent was added to each well. Luminescence was recorded after 60 min of incubation at room temperature, and NAD^+^ to NADH ratio was calculated (SpectraMax ID3 Multi-Mode Microplate Reader, Molecular Devices, LLC, San Jose, CA, USA).

### Determination of ATP production rate

The ATP production rate, oxygen consumption rate (OCR), and extracellular acidification rate (ECAR) were measured using the Agilent Seahorse ATP Real-Time rate assay kit (Agilent, Santa Clara, CA, USA) on a Seahorse XFp analyzer (Agilent, Santa Clara, CA, USA) according to the manufacturer's instructions. Briefly, wells were coated with 20 µL of Cell-Tak (22.4 µg/mL, Corning). Cells were seeded at 37500 cells per well. Before measurement, plates were equilibrated in a CO_2_-free incubator for 1 h. The analysis used 1.5 µM oligomycin and 0.5 µM rotenone/antimycin A. Data were analyzed using Seahorse XF Real-Time ATP rate assay report generator software (Agilent, Santa Clara, CA, USA).

### Docking studies

PatchDock server and AutoDock4 program were used in the docking simulation studies. The docking structure template of human PARP1 with double-strand DNA was obtained from Protein Data Bank (PDB ID: 4OQA), and the coordinates of 3ABA, MAC681, 3ABA-MAC681-5-position, and 3ABA-MAC681-6-position adducts compounds were created using the ChemDraw program. To initially predict the binding mode of MAC681, we implemented the simulation docking using PatchDock webserver with PARP1 used as a receptor. After coarse prediction of docking positions and scores according to the geometric shape complementarity in PatchDock, the top 20 docking position candidates were further analyzed. To further refine the binding mode of our interest compounds, we ran the AutoDock4 program with a flexible residues option for Asp766, His862, Tyr889, Phe897, Gly894, Ser904, Tyr907, and Glu988, and those residues have been shown to contribute to ligand binding. Firstly, a control docking experiment with a known ligand of PARP1, 2,3-dihydrobenzofuran-7-carboximade derivative (2US) was done by AutoDock4 to re-confirm the binding mode with PARP1. Structural alignment and docking figures were created by the PyMOL program.

### Mass spectrometry

K-562 cells (1.2 × 10^8^) were seeded in 50 ml of media (DMEM plus 10% FBS) containing 25 mM 3ABA. After 30 min incubation at 37˚C, 25 µM MAC681 was added, and the cells were incubated for an additional 6 h at 37˚C. Following incubation with 3ABA and MAC681, the cells were collected and pelleted by centrifugation. The cell pellet was washed 2 times with 1 × PBS and then solubilized by adding 0.5 ml of ice-cold acetonitrile. The sample was centrifuged at 13000 g for 3 min (4˚C), after which the supernatant was collected. The supernatant was dried under vacuum at 45˚C for 1 h and then resuspended in 20 µl of acetonitrile. Samples (5 µl) were then separated by reverse-phase HPLC and then analyzed by mass spectrometry using an Agilent 6410 triple quadrupole mass spectrometer (Santa Clara, United States).

### ALDH enzymatic assay

Aldehyde dehydrogenase (ALDH) enzymatic activity was assessed using the Aldefluor™ kit (StemCell Technologies Inc., Saint Égrève, France) according to the manufacturer’s instructions. Diethylaminobenzaldehyde, a specific inhibitor of ALDH activity, was used to differentiate cells with low and high ALDH activity. Stained samples (50,000 events/sample) were recorded by flow cytometry (FACS LSR Fortessa™ X-20, BD Biosciences). Non-viable cells were excluded from analysis following staining with propidium iodide (BD Biosciences).

### Statistical analysis

Data are expressed as the mean ± SD of at least 3 independent experiments, and significance was estimated by t-test, one-way or two-way ANOVA using Prism 9 software, GraphPad Software (La Jolla, CA, USA). Additionally, we evaluated the Area Under the Curve (AUC) for specific datasets using GraphPad Prism 10. The AUC analysis provided a comprehensive assessment of the overall response magnitude over the given time ranges. *P*-values < 0.05 were considered significant.

## Results

### Identification of an altered metabolism and DNA repair transcriptomic signature in CML patients and cell lines

**Fig. 1:**
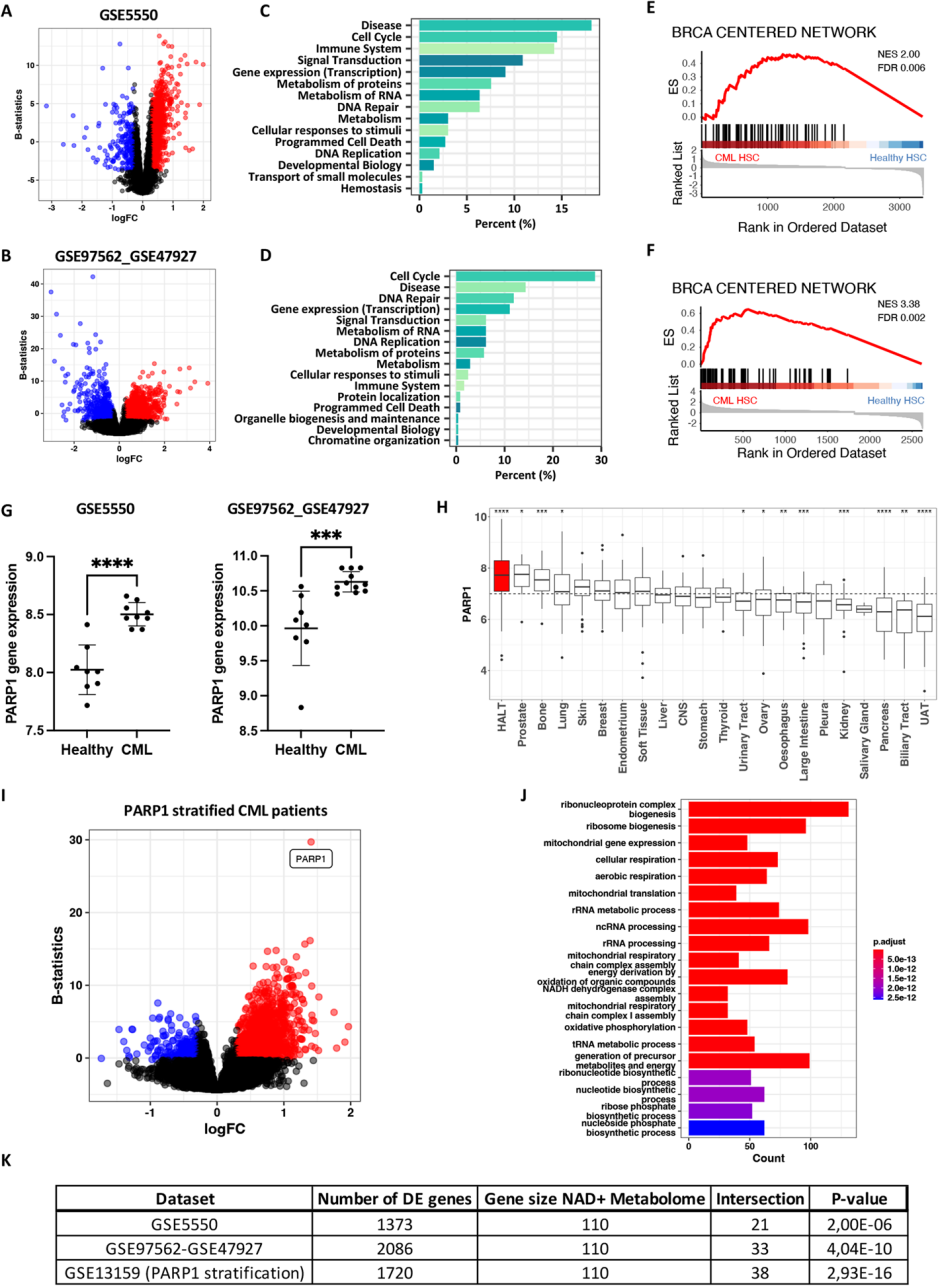
Identification of an altered metabolism and DNA repair transcriptomic signature in CML patients and cell lines. **A**, **B** Volcano plot showing genes that were significantly dysregulated in CML CD34^+^ (|log_2_FC|≥ 0.3; false discovery rate (FDR) ≤ 0.05) compared to healthy donors in GSE5550 [**A**] and combined GSE97562_GSE47927 [**B**]. DEGs significantly upregulated are represented in red, and DEGs significantly downregulated in blue. **C**, **D** Bar plots of all Reactome pathways significantly altered in CD34^+^ CML samples compared with the normal counterpart summarized as a percent in the mother category to the total number of enriched pathways in GSE5550 [**C**] and combined GSE97562_GSE47927 [**D**] (corrected for the number of pathways annotated by category). **E**, **F** GSEA plots of the gene signature linked to BRCA mutated tumors proposed by Pujana et al. [35] in GSE5550 [**E**] and combined GSE97562_GSE47927 [**F**]. **G** CML patient stem cells overexpress PARP1 compared to stem cells of healthy volunteers in indicated datasets (GSE5550 and combined GSE47927 with GSE97562, double-sided unpaired t-test ***p ≤ 0.001, ****p ≤ 0.0001). **H** PARP1 gene expression profiles in different cancer cell lines categorized by tissue origin from the CCLE dataset. CML and AML cell lines are included in the group “Hematopoietic and lymphoid tissue (HALT)”, the group with the highest PARP1 gene expression (Mann–Whitney test to the overall mean, *p ≤ 0.05, **p ≤ 0.01, ***p ≤ 0.001, ****p ≤ 0.0001). CNS: central nervous system, UAT: upper aerodigestive tract. **I** Volcano plot representing the genes significantly differentially expressed (|log_2_ fold change (FC)|≥ 0.3; FDR ≤ 0.05) in CML patients divided into top and lower quartiles of PARP1 expression (GSE03159, 66 patients). DEGs significantly upregulated are represented in red, and DEGs significantly downregulated in blue. **J** Top 20 biological gene ontology terms significantly enriched in patients with high PARP1 expression. This analysis highlights enrichment in multiple biological processes linked to mitochondria functions. **K** Table of genes enriched in NAD^+^ metabolome (Fisher’s exact test)

We investigated genes differentially expressed in CML compared to healthy CD34^+^ hematopoietic stem and progenitor cells in publicly available data sets (combined GSE47927_GSE97562 and GSE5550, Supp. document SD1A), focusing on untreated patients in the chronic phase of the disease. The differential expression analysis revealed 1371 DEGs in GSE5550 (1169 up, 202 down, |log_2_FC|> 0.3; false discovery rate [FDR] ≤ 0.05) (Fig. 1A, Supp. document SD1B) and 2053 DEGs in combined GSE47927_GSE97562 (1532 up, 521 down, |log_2_FC|> 0.3; FDR ≤ 0.05) (Fig. 1B, Supp. document SD1C). The DEGs showed a significant overlap between the datasets (Fisher’s exact test p-value = 8.3e-77, odds ratio = 3.9), indicating a strong correlation between the datasets. As a first global approach, we investigated the DEGs by performing a Reactome pathway enrichment analysis (Fig. 1A-B, Supp. document SD1D-E, Supp. Fig. S1A-B). Both datasets were significantly enriched in pathways linked to cell cycle, disease, immune system, gene expression, signal transduction, DNA replication, DNA repair, and multiple categories linked to metabolism (Fig. 1A-B, Supp. document SD1D-E). As previously reported, these results suggest that primary CML stem and progenitor cells of chronic phase CML patients present an aberrant proliferation with an increased metabolism to sustain high energetic demands [34]. Interestingly, pathways in DNA repair and disease, including DNA repair defects, were highly represented in both datasets, including diseases of mismatch repair and double-strand break repair linked to BRCA1/2 loss of function (Fig. 1C-D, Fig. S1A-B, Supp. document SD1D-E).

We performed a GSEA of the gene signature linked to BRCA-mutated tumors proposed by Pujana et al. [35] (Fig. 1E-F). CML datasets showed enrichment in this network, suggesting that CML patients are deficient in double-strand break repair pathways. We decided to investigate the status of PARP1 in CML, as PARP1 inhibitors have been approved as treatments for cancer types with deficient DNA repair [36, 37]. A comparative analysis of gene expression profiles in CML patient datasets (GSE5550 and GSE47927_GSE97562) showed that PARP1 is significantly upregulated in CML patients’ stem cells compared to healthy counterparts (Fig. 1G). Moreover, using the CCLE (Fig. 1H), we found that hematopoietic and lymphoid tissues cell lines present the highest PARP1 gene expression levels when compared to all other cancer (Wilcoxon test to the overall mean, p ≤ 2e-16). The median log_2_TPM in the myeloid subgroup in hematopoietic and lymphoid tissues was 7.41 (AML) and 7.36 (CML). Interestingly, the mean expression was higher than in other cancer types, including cancers where the utilization of PARP1 inhibitors was approved or under clinical evaluation, including ovarian, breast, pancreatic, and prostate cancers [38, 39]. This observation further substantiates using PARP1 as a target in hematopoietic and lymphoid cancers. We further verified the correlation between gene and protein expression in the CCLE dataset. PARP1 presents a good correlation between mRNA and protein expression (Pearson’s r 0.67 and Spearman’s r 0.65), justifying using PARP1 gene expression as a proxy of the protein.

To investigate biological shifts associated with PARP1, we compared the whole-genome expression profiles of patients with high PARP1 expression and patients with low PARP1 expression at diagnosis (GSE13159, 66 CML patients’ mononuclear cells of the bone marrow). We identified a set of 1720 genes differentially expressed (1528 up and 192 down, |log_2_FC|> 0.3; FDR ≤ 0.05) (Fig. 1I and Supp. document SD1F). Gene ontology terms analysis (Fig. 1J) showed a clear association of genes overexpressed in high PARP1 patients in processes linked to mitochondrial function, including mitochondrial transcription, translation, and cellular respiration, in particular, complex I (NADH dehydrogenase complex). It indicates increased dependence on mitochondrial activity and OXPHOS for NAD^+^ replenishment.

To further investigate if patients' CML stem cells were also enriched in such pathways, we assessed enrichment in genes involved in the “NAD^+^ metabolome” category [10]. These genes included the NAD^+^ salvage pathway, NAD^+^ de novo pathways, complex I, TCA cycle, and aerobic glycolysis. Our results show that CML stem cells were enriched in such pathways compared to their healthy counterparts (Fig. 1K Supp. document SD1G, all *p* < 0.05 Fisher Exact test).

### MAC681 induces DNA damage, mitochondrial dysfunction, perturbation of calcium homeostasis, and metabolic catastrophe

**Fig. 2:**
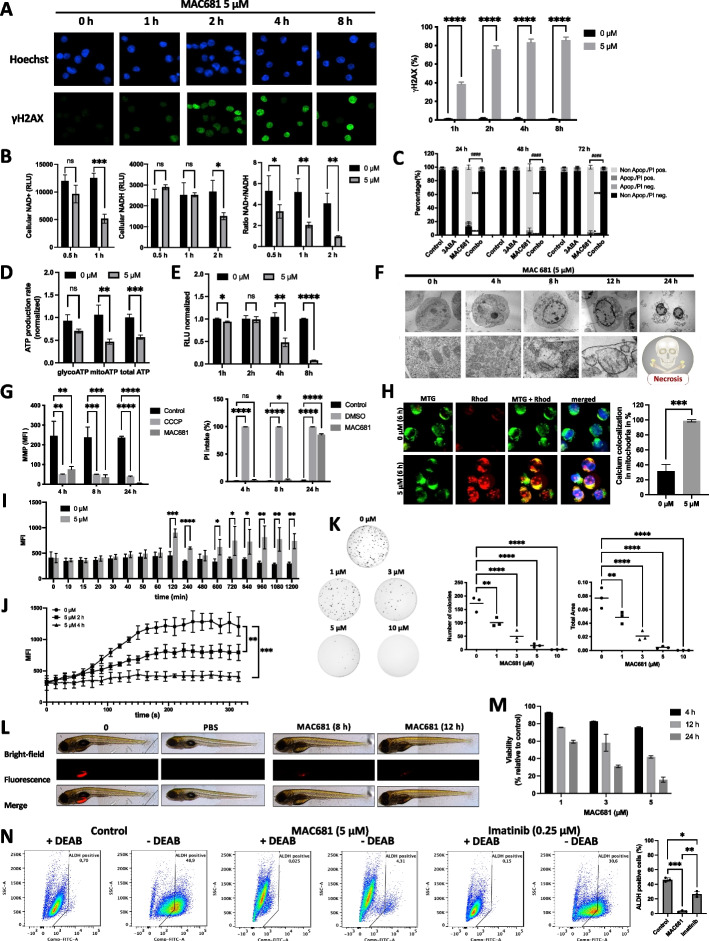
MAC681 induces DNA damage and mitochondrial dysfunction, leading to metabolic catastrophe. K-562 cells were treated with MAC681 at indicated time points and concentrations. **A** The formation of γH2AX nuclear foci was assessed by fluorescent microscopy (left panel) and quantified by flow cytometry (right panel) (double-sided unpaired t-test ****p ≤ 0.0001). **B** Total oxidized NAD^+^ (left panel) and the reduced NADH (middle panel) was detected by bioluminescent NAD/NADH-Glo™ Assay, and NAD^+^/NADH ratios (right panel) were determined in MAC681-treated cells at the indicated time points, (double-sided unpaired t-test *p ≤ 0.05, **p ≤ 0.01, ***p ≤ 0.001, ****p ≤ 0.0001). **C** Quantification of the indicated cell populations after Hoechst/PI staining in cells treated with MAC681 (5 µM) with or without 3ABA (5 mM) 30 min pre-treatment (one-way ANOVA, Šídák's multiple comparisons test *p ≤ 0.05, ****p ≤ 0.0001, ####*p* ≤ 0.0001). **D** The basal ATP production rates from glycolysis and mitochondrial respiration and total ATP production rates were measured by Seahorse XF Real-Time ATP Rate Assay Kit in K-562 cells 2 h after exposure to MAC681 (5 µM) (double-sided unpaired t-test **p ≤ 0.01, ***p ≤ 0.001). **E** Intracellular ATP content of MAC681 (5 µM)-treated K-562 cells was measured by CellTiter-Glo at indicated time points (double-sided unpaired t-test *p ≤ 0.05, **p ≤ 0.01, ****p ≤ 0.0001). **F** TEM of K-562 cells treated with MAC681 (5 µM) at indicated time points. Morphological changes of cells: extensive vacuolization of the cytoplasm of the dead cell and an extreme dilatation of the perinuclear space impinging on the nucleus (24 h), a hallmark of necroptosis, are seen (top panel). Mitochondrial alterations (lower panel) induced by MAC681 are also visualized. **G** Time-dependent induction of mitochondrial membrane potential loss (left panel) and cell death induction (right panel) by MAC681 (5 µM). MMP was analyzed after incubation with MitoTracker Red (100 nM) by flow cytometry. Mitochondrial oxidative phosphorylation uncoupler CCCP (50 µM, 20 min) was used as a positive control. Flow cytometry analyzed cell death induction by propidium iodide (PI) uptake after 15 min staining with PI (0.5 µg/mL). DMSO (50%, 1 min) was used as a positive control (one-way ANOVA, Dunnett's multiple comparisons test *p ≤ 0.05, **p ≤ 0.01, ***p ≤ 0.001, ****p ≤ 0.0001). **H** Cells were treated with MAC681 (5 µM, 6 h) and analyzed by fluorescence microscopy after staining with Rhod2-AM (Rhod, 2.5 µM), MitoTracker Green (MTG, 100 nM), and Hoechst. The intensity of the Rhod2-AM signal was quantified in regions stained with MitoTracker Green, and the co-occurrence and correlation were quantified by Manders’, Spearman’s, and Pearson’s coefficients. Double positive cells were analyzed, and the average of three independent experiments was shown (double-sided unpaired t-test ***p ≤ 0.001). **I** Cytosolic Ca^2+^ levels of cells treated with MAC681 (5 µM) for indicated time points, stained by Fluo4-AM, were measured by flow cytometry (double-sided unpaired t-test *p ≤ 0.05, **p ≤ 0.01, ***p ≤ 0.001, ****p ≤ 0.0001). **J** Cytosolic Ca^2+^ levels of cells treated with MAC681 (5 µM) for indicated time points in the presence of EGTA (650 µM), followed by TSG (10 nM) treatment, were measured by Fluo4-AM every 15 s by flow cytometry, (AUC**,** double-sided unpaired t-test **p ≤ 0.01, ***p ≤ 0.001). **K** Colony formation assay with K-562 cells treated with MAC681 at the indicated concentrations. Images are representative of three independent experiments (One-way ANOVA, Dunnett's multiple comparisons test **p ≤ 0.01, ***p ≤ 0.001, ****p ≤ 0.0001). **L** In vivo zebrafish xenografts model of tumor mass formation after injection of fluorescent CellTracker™ CM-DiI Dye-stained K-562 cells pre-treated for the indicated time. **M** MAC681 decreases the viability of CML patients’ PBMCs in dose- and time-dependent manners. CML primary cells were treated *ex-vivo* with indicated concentrations of MAC681, and cell viability was evaluated after 4, 12, and 24 h by trypan blue exclusion test. The histogram corresponds to the mean ± SD (*n* = 2 CML patients). **N** Analysis of ALDH activity in K-562 cells. Cells were treated with MAC681 (5 µM, 12 h) or Imatinib (0.25 µM, 24 h), and the ALDH inhibitor diethylaminobenzaldehyde (DEAB) was used to distinguish cell subpopulations with low and high ALDH activity. Representative dot plots showing the percentage of ALDH-positive cells and corresponding quantifications (right panel) of three independent experiments are presented (left panel) (one-way ANOVA, Tukey’s multiple comparisons test *p ≤ 0.05, **p ≤ 0.01, ***p ≤ 0.001)

We considered the deregulation of gene expression patterns in CML patients (Fig. 1) as a vulnerability that DNA-damaging agents can target. We systematically monitored DNA damage induction through phosphorylation of histone H2AX at Ser 139 (γH2AX), a marker of DNA double-strand breaks. As shown in Fig. 2A, MAC681 induced an early, time-dependent appearance of γH2AX-positive foci in 38.3% of the cell population after 1 h and reached a plateau of 76—86% positive cell population between 2 to 8 h.

Considering the upregulation of PARP1 in CML patients' datasets and the upregulation of metabolic pathways in patients expressing high levels of PARP1, we wondered if such a strong induction of DNA damage could result in the PARP-dependent depletion of total cellular NAD^+^ levels. Indeed, after MAC681 treatment, we detected an early, time-dependent decrease in cellular NAD^+^. MAC681 treatment reduced NAD^+^ by 20% in 30 min, 59% in 1 h, and 87% in 2 h. NAD^+^ depletion was followed by a reduction in NADH levels. An overall time-dependent decrease in the NAD^+^/NADH ratio was detected (Fig. 2B). Moreover, Hoechst/PI nuclear morphology analysis indicated that MAC681 induced necrotic cell death in K-562 cells and that the PARP1 inhibitor, 3ABA rescued cells from MAC681-induced lethality (Fig. 2C).

As metabolic pathways, including OXPHOS, rely on NAD availability, PARP1 activation promotes a rapid decrease in mitochondrial oxygen consumption [40]. We detected a significantly decreased OCR in MAC681-treated cells compared to the control (Suppl. Fig. S2A). Moreover, in K-562 cells, the basal ATP production rate relied more on oxidative phosphorylation than glycolysis (61% versus 39%, respectively) (Sup. Fig. S2B). Within two hours, MAC681 treatment induced a 43% reduction in the total ATP production rate compared to untreated cells (Fig. 2D). Furthermore, the MAC681-induced decrease in the ATP production rate was mainly due to decreased mitochondrial ATP production rate (Fig. 2D). Subsequently, after 4 h of treatment, we observed a 53.2% reduction in total intracellular ATP levels. After 8 h, ATP levels were reduced by 92% compared to controls (Fig. 2E).

The loss of NAD^+^ precedes the induction of mitochondrial depolarization and mitochondrial outer membrane permeability transition [41]. TEM analysis confirmed time-dependent mitochondrial alterations after MAC681 treatment, disrupting mitochondrial cristae after 4 h and mitochondrial swelling after 8 h (Fig. 2F, lower panel). In addition, the cells adopted a necroptotic phenotype (Fig. 2F, upper panel). In agreement with morphological changes and ATP loss, MAC681 triggered a time-dependent loss of mitochondrial membrane potential (MMP) (Fig. 2G, left panel). The MMP was strongly reduced at 4 h and 8 h of treatment, although cells remained viable, considering the minimal PI staining intake (Fig. 2G, right panel).

Since mitochondrial calcium overload could have triggered these mitochondrial morphological alterations, we assessed calcium accumulation in mitochondria by co-localizing Rhod2-AM mitochondrial Ca^2+^ dye with MitoTracker green after 6 h of treatment. Our results indicate that MAC681 induced Ca^2+^ accumulation in 67% of the mitochondria (Fig. 2H and Sup. Fig. S2C). To further analyze the perturbation of calcium homeostasis, we assessed the effect of MAC681 on cytosolic Ca^2+^ accumulation after Fluo4-AM staining. We detected an immediate increase in cytosolic Ca^2+^ at 2 h, which decreased after 4 h and returned to basal values at 8 h. At 10 h, we observed a secondary increase in cytosolic Ca^2+^ (Fig. 2I).

To further ascertain the origin of the observed cytosolic Ca^2+^ accumulation, we used EGTA to chelate extracellular Ca^2+^ influx. Under these conditions, adding the sarcoplasmic/endoplasmic reticulum Ca^2+^-ATPase (SERCA) inhibitor thapsigargin (TSG) led to a rapid accumulation of cytosolic Ca^2+^ in control cells. Interestingly, in MAC681-treated cells for 2 h, the addition of TSG significantly reduced the increase, which was abolished at 4 h (Fig. 2J). These data show that MAC681 rapidly depleted endoplasmic reticulum Ca^2+^ in line with the observed initial cytosolic Ca^2+^ increase.

PARP1 activation after DNA damage contributes to mitochondrial Ca^2+^ dysregulation and subsequent calpain activation [42, 43] to promote truncation and translocation of apoptosis-inducing factor (AIF) from the mitochondria to the nucleus. In MAC681-treated cells, AIF co-localized with nuclear Hoechst staining (Sup. Fig. S2D-E) compared to untreated controls. Moreover, we observed that calpain inhibitor PD 150606 prevented the PARP1 degradation (Sup. Fig. S2F), suggesting the involvement of calpain activation during MAC681-induced metabolic catastrophe.

### The translational potential of MAC681 as an antileukemic agent in vitro and in vivo

Considering the observed antimetabolic potential of the methylated indolequinone MAC681, we assessed its effects on the viability in four human leukemia cells (K-562, K-562R, Jurkat, and U-937) (Sup. Fig. S2G). To assess differential toxicity, we treated the non-cancerous, proliferating cell line RPMI1788 representing healthy B lymphocytes and PBMCs obtained from healthy donors with increasing concentrations of MAC681. RPMI 1788 cells were 65-fold and PBMCs 51-fold less affected compared to K-562 cells when treated at 10 µM after 72 h (Sup. Fig. S2H).

Considering the above-mentioned antileukemic effect of MAC681 at low µM concentrations, we investigated the effect of MAC681 by CFA. Results showed a dose-dependent decrease in the colony formation capacity of three chronic myeloid leukemia cell lines (K-562, KBM-5, Meg-01) and two acute myeloid leukemia cell lines (U-937, HL-60) (Fig. 2K and Sup. Fig. S2I-J). Next, we extended CFA results toward a zebrafish xenograft model to provide a more solid basis for the translational value of MAC681. In vivo data showed time-dependent inhibition of tumor mass formation (Fig. 2L and Sup. Fig. S2K). Moreover, we treated *ex-vivo* primary CML cells from two CML patients with MAC681. As seen in Fig. 2M, the MAC681 efficiently reduced the viability of CML patients’ PBMCs in dose- and time-dependent manners. We next investigated if MAC681 could target ALDH-positive cells. High expression of ALDH has been reported for normal and cancer stem and progenitor cells of various lineages, including hematopoietic cells [44, 45]. Elevated ALDH activity is an established marker for the identification of hematopoietic cells with stem-like characteristics. Our data shown in Fig. 2N provide evidence that MAC681 (5 µM, 12 h) is targeting preferentially the ALDH-positive population, decreasing the amount of ALDH bright cells by 93.3%. Interestingly, imatinib treatment (0.25 µM) at 24 h reduced the ALDH-positive population only by 42.4%. Our data validated MAC681 as an attractive antileukemic agent for further mechanistic investigations.

### Off-target prevention of MAC681-induced cell death by the PARP inhibitor 3-aminobenzamide

**Fig. 3:**
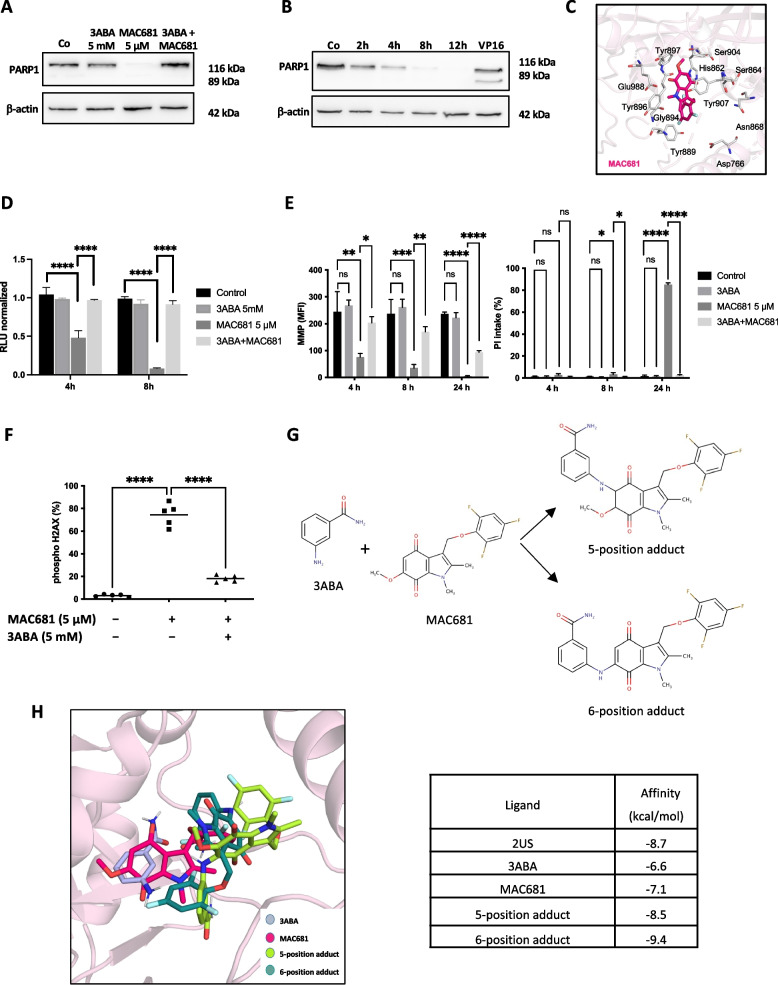
Off-target protective effect of 3ABA in MAC681-treated cells. **A** Western blot analysis of PARP1 protein levels in K-562 cells treated with MAC681 (5 µM), 3ABA (5 mM), or their combination after 4 h. **B** Kinetic analysis of the PARP1 protein levels in K-562 cells treated with MAC681 at 5 µM. β-actin was used as a loading control. Etoposide 100 µM (VP16) was used as a positive control. **C** Closed-view binding mode of MAC681 in the catalytic pocket of PARP1. The well-known catalytic residues of PARP1 are presented in a white stick model with nitrogen, oxygen, and fluorine colored blue, orange, and cyan, respectively. MAC681 is presented as a magenta stick. **D** Intracellular ATP content in K-562 cells treated with MAC681 (5 µM), 3ABA (5 mM), or their combination normalized to control cells measured by CellTiter-Glo at indicated time points (one-way ANOVA, Tukey's multiple comparisons test ****p ≤ 0.0001). **E** Effects of MAC681 (5 µM), 3ABA (5 mM), or their combination on mitochondrial membrane potential (MMP) analyzed by flow cytometry with MitoTracker Red staining (left panel) at indicated time points. MFI represents median fluorescence intensity. Cell death was assessed simultaneously with propidium iodide (right panel) (one-way ANOVA, Šídák's multiple comparisons test *p ≤ 0.05, **p ≤ 0.01, ***p ≤ 0.001, ****p ≤ 0.0001). **F** Quantification of γH2AX nuclear foci formation by flow cytometry in K-562 cells treated with MAC681 (5 µM), 3ABA (5 mM), or their combination (one-way ANOVA, Dunnett's multiple comparisons test ****p ≤ 0.0001). Results correspond to the mean ± SD of three independent experiments. **G** Chemical structures of MAC681 and 3ABA are recognized as suitable reaction partners that can give rise to 5-position or 6-position adducts. **H** The 5-position and 6-position adducts bind to PARP1 in the same mode as 3ABA and MAC681 (left panel). Stabilization energies of docking compounds to PARP1 are shown (right panel). Compound 2US was used as a control for docking experiments

As we previously detected a protective effect of the PARP inhibitor 3ABA against MAC681-induced cell death (Fig. 2C), we next assessed the effect of the 3ABA pre-treatment on the PARP1 protein level. Surprisingly, we observed the disappearance of full-length PARP1 (116 kDa) without any cleaved fragments after 8 h of MAC681 treatment alone. 3ABA pre-treatment prevented this PARP1 reduction (Fig. 3A). Hence, we analyzed the kinetics of the atypical MAC681-induced PARP1 reduction (Fig. 3B). We used an additional antibody able to recognize both apoptotic as well as necrotic PARP1 cleaved fragments (Supp. Fig. S3A). However, we did not detect any cleavage fragments either. In addition, we observed that MAC681 induced a time-dependent downregulation of PARP1 mRNA expression levels, which is prevented in the presence of 3ABA (Supp. Fig. S3B). Next, we verified whether the disappearance of full-length PARP1 might be related to its low mRNA levels after MAC681 treatment. Accordingly, we analyzed the stability of PARP1 in the presence of cycloheximide that blocks the elongation phase of protein synthesis (Supp. Fig. S3C). We showed that the stability of PARP1 protein remains unchanged up to 24 h in the presence of cycloheximide, proving that the PARP1 protein degradation observed after MAC681 treatment is independent of its effects on PARP1 mRNA levels.

This type of atypical degradation could be explained by the direct interaction of MAC681 with PARP1 protein, a hypothesis we validated by computational docking. The control docking showed that 2, 3-dihydrobenzofuran-7-carboximade derivative (2US) was docked into the catalytic region of PARP1 in good agreement location with the actual binding mode in the crystal structure of PARP1 in complex with 2US (PDB ID: 40QA) (Supp. Fig. S3D, upper panel). We demonstrated that MAC681 binds with high affinity into the catalytic region of the PARP1 structure in the presence of damaged DNA (Supp. Fig. S3D, lower panel, MAC681 in magenta). MAC681 was located in active sites of the PARP1 with stabilization energies of − 7.1 kcal/mol. We closely observed that the aromatic ring of the bound MAC681 is surrounded by well-known inhibitor-binding residues such as His862, Tyr889, Tyr896, Phe897, and Tyr907 (Fig. 3C).

We further focused on the protective role of 3ABA. We assessed its effects on MAC681-induced ATP depletion and mitochondrial membrane potential (MMP) loss leading to cell death as analyzed by PI intake. 3ABA alone did not affect cellular ATP levels. On the other hand, 3ABA pre-treatment restored ATP levels that were depleted after the MAC681 treatment (Fig. 3D). Similarly, 3ABA alone did not affect MMP. It partially reverted the loss of MMP compared to cells treated with MAC681 alone (Fig. 3E left panel). Moreover, 3ABA rescued the cells from dying, as shown by PI intake (Fig. 3E right panel).

3ABA prevented the DNA damage induced by MAC681 not only in K-562 cells (Fig. 3F) but also in U-937, MOLM-13, MV4-11, and OCI-AML3 cell lines (Supp. Fig. S3E left panel) where MAC681 induced typical apoptotic nuclear fragmentation patterns (Supp. Fig. S3E right panel). 3ABA prevented observed nuclear morphology changes in all cell models. Concomitantly, 3ABA counteracted the characteristic apoptotic fragmentation of PARP1 protein caused by MAC681 treatment in U-937 cells (Supp. Fig. S3F). Interestingly, this protection (of cell death induction and PARP1 fragmentation) by 3ABA could not be observed in apoptotic U-937 cells treated with etoposide (Supp. Fig. S3F), demonstrating the specificity of a combination involving MAC681 and 3ABA. These data indicate that the protective effect of 3ABA in MAC681 treated cells could be independent of its primary PARP1-inhibition function and instead mediated by its so far not-described off-target effect.

The chemical structures of MAC681 and 3ABA are recognized as suitable reaction partners. Based on our previous results, the chemical structure of 3ABA seemed to be directly involved in the protective effect. Thus, we hypothesized that indolequinone MAC681 could act as a Michael acceptor, bearing the α,β-unsaturated bond, reacting with the aniline nucleophile of 3ABA (Michael donor), able to attack the position next to the -OCH_3_ of the quinone ring of MAC681. The above-mentioned Michael reaction would result in a hypothetical 3ABA-MAC681-5-position adduct (Fig. 3G). On the other hand, the reaction between 3ABA and MAC681 can also give rise to the 6-position adduct due to the loss of the methoxy group with the addition of the amine and the 2,4,6-trifluorophenol part of the molecule either intact (Fig. 3G) or lost (not shown). Using mass spectrometry, we confirmed the presence of a 6-position adduct when cells were treated with a combination of MAC681 and 3ABA for 6 h (Supp. Fig. S3G). Moreover, the 3ABA washout completely abrogates the protective effect of 3ABA detected without washout (Suppl. Fig. S3H). In the washout group, cells pre-treated with 3ABA and treated with MAC681 for 4 h were losing ATP at the same extent as cells treated with MAC681 alone (S3H upper left panel). The loss of the protection by 3ABA on early effects mediated by MAC681 was confirmed by the late outcome on cell concentration (S3H upper middle panel) and viability (S3H upper right panel). In addition, we confirmed that the cells in the washout group (3ABA + MAC681) were dying by the same cell death modality as cells treated with MAC681 alone, as shown by identical nuclear morphology patterns (S3H lower panel).

Indolequinones' DNA damaging potential is linked to their redox-cycling activity or alkylation potential. Using dichlorofluorescein and redox sensor red probes, we confirmed that MAC681 did not induce any reactive oxygen species (ROS) (Supp. Fig. S3I) and thus did not act as a redox cycler. Considering its alkylation activity, MAC681 could be transformed into a DNA alkylation agent through bioreductive activation by two electron reductases, such as NAD(P)H quinone dehydrogenase (NQO)1 or NQO2, via mechanisms of iminium salt (Supp. Fig. S3J) [46] or quinone methide formation (Supp. Fig. S3K) [47]. Analysis of CML patient datasets showed that CML patients have significantly higher expression of NQO1 or NQO2 or both than their healthy counterparts (Supp. Fig. S3L). Moreover, based on the CCLE dataset, K-562 cells have the highest expression of NQO2 and the second highest expression of NQO1 within the CML cell lines available (Supp. Fig. S3M).

Considering this, the above-mentioned hybrid compounds (5-position and 6-position adducts) would reduce the overall DNA damaging activity of MAC681 and thus confer 3ABA-mediated protection against MAC681-induced controlled necrosis triggered by excessive DNA damage.

In addition, docking results revealed that 3ABA and both hybrid compounds (3ABA-MAC681-5-position adduct and 3ABA-MAC681-6-position adduct) docked well into the similar PARP1 catalytic region where MAC681 did. Interestingly, both hybrid compounds have bound to PARP1 tighter than MAC681 or 3ABA alone, with stabilization energies of -9.4 and -8.5 kcal/mol, respectively (Fig. 3H).

### MAC681-induced cell death has immunogenic potential

**Fig. 4:**
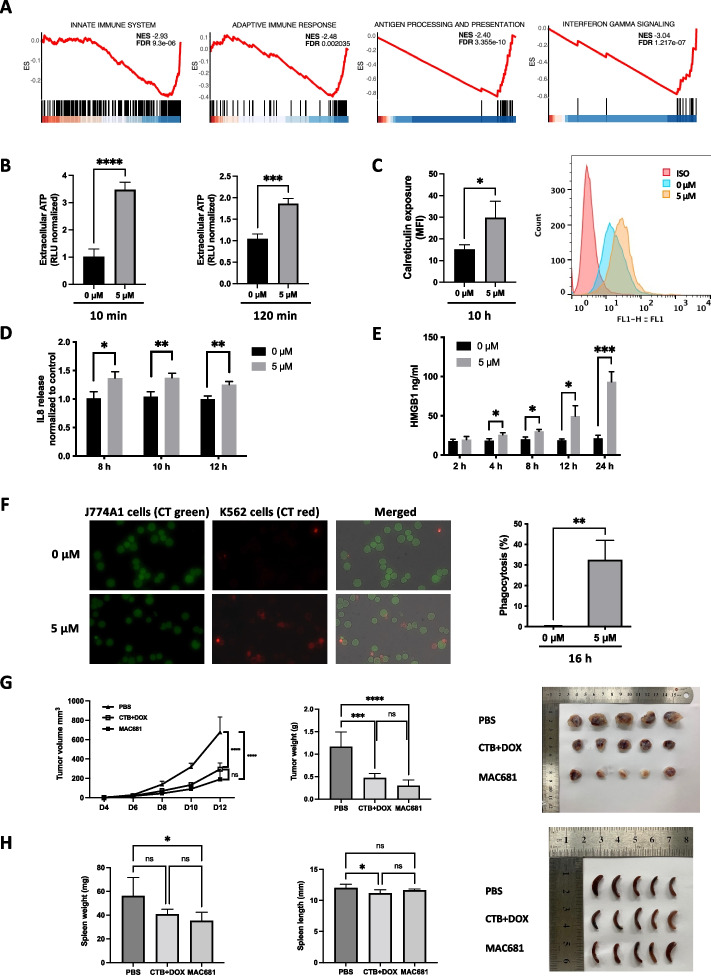
MAC681 has an immunogenic potential in myeloid disorders. **A** GSEA plots showing genes linked to innate and adaptive immune response, antigen processing and presentation, and interferon-gamma signaling have negative NES scores in CML CD34^+^ cells (ES: enrichment Score, NES: normalized enrichment score). **B** Extracellular release of ATP into the supernatant induced by 5 µM of MAC681 at indicated time-points in K-562 cells was measured in a luciferase-based assay (double-sided unpaired t-test ***p ≤ 0.001, ****p ≤ 0.0001). **C** Ectopic calreticulin expression in K-562 cells was investigated by flow cytometry after 10 h of MAC681 treatment at 5 µM. ISO corresponds to isotypic control. Quantification (left panel) and representative experiment (right panel) (double-sided unpaired t-test *p ≤ 0.05, MFI = median fluorescence intensity). **D** Time-dependent release of IL-8 induced by MAC681 at 5 µM assessed by ELISA (double-sided unpaired t-test *p ≤ 0.05, **p ≤ 0.01). **E** Time-dependent release of HMGB1 after treatment with 5 µM of MAC681 assessed by ELISA in K-562 cells supernatants (double-sided unpaired t-test *p ≤ 0.05, ***p ≤ 0.001). **F** Phagocytosis of MAC681-treated (5 µM, 16 h) red-stained K-562 CML cells by green-stained J774A1 murine macrophages assessed by fluorescent microscopy. Murine macrophages were co-cultured with CML cells for 2 h. Representative pictures are shown on the left, and quantification on the right (double-sided unpaired t-test **p ≤ 0.01). **G** Kinetic analysis of tumor volume (left panel) in syngeneic immunocompetent C57BL/6 mice and the quantification of the overall response using the AUC. Tumor weight (middle panel) was measured after sacrifice, and excised tumors were visualized (right panel). “D” corresponds to days after inoculation with living C1498 cells (one-way ANOVA, Tukey's multiple comparisons test ***p ≤ 0.001, ****p ≤ 0.0001A). **H** Spleen weight (left panel) and spleen length (middle panel) were assessed after mice sacrifice (one-way ANOVA, Tukey's multiple comparisons test *p ≤ 0.05). Pictures of spleen excised from C57BL/6 mice are visualized (right panel). Error bars represent the SD of at least three independent experiments

Our initial analysis of transcriptomic data (GSE5550) showed a significant down-regulation of genes encoding proteins involved in adaptive and innate immune responses in CML patients (Fig. 4A). Especially genes leading the NES score, belonging to antigen processing and presentation and interferon-gamma signaling categories are down-regulated (Fig. 4A). As the attenuation of these essential immune recognition mechanisms reduces the capacity of the patient’s immune effectors to recognize and eliminate the accumulating CML blasts, we were interesting to characterize further the MAC681-induced necrotic cell death observed in K-562 cells from the immunogenic perspective. We measured the emission of ICD-related DAMPs, including calreticulin exposure, extracellular ATP secretion, and HMGB1 release. As soon as 10 min and up to 2 h after the treatment, MAC681-treated cells reached between 2- to fourfold increase in ATP secretion compared to untreated controls (Fig. 4B). Additionally, we determined that 10 h of 5 µM MAC681 treatment doubled the exposure of CRT at the cell surface compared to the control (Fig. 4C). Besides intrinsic cellular mechanisms, the translocation of calreticulin is also mediated by soluble factors that operate in an autocrine/paracrine manner. Accordingly, IL-8 can be hyperactivated by ICD-inducers to facilitate the immunogenic exposure of calreticulin [48]. Hence, we measured the human IL-8 concentrations in cell culture supernatants and showed that 5 µM of MAC681 significantly induced IL-8 release at 8 and 10 h of treatment compared to control (1.4- and 1.3-fold increase, respectively) (Fig. 4D). Timeframe of IL-8 release is in line with calreticulin cell surface exposure in K-562 cells. In addition, we also provided evidence showing that at 8 h, MAC681 induced IL-8 mRNA expression in a dose-dependent manner in K-562 cells with a 6.4-fold increase at 5 µM (Supp. Fig. S4A).

Furthermore, we showed that necrosis-inducing concentration triggered significant HMGB1 release starting from 4 h and peaking at 24 h in MAC681-treated K-562 cells (Fig. 4E). Moreover, we showed that MAC681-induced HMGB1 release not only in CML cells but also in OCI-AML3 and C1498 AML cell lines (Supp. Fig. S4B-C). As the emission of DAMPs by dying cells plays a crucial role in recruiting immune effectors, we co-cultured J774A1 macrophages with MAC681 pre-treated K-562 cells. Fluorescence microscopy showed a 160-fold increase in the engulfment of red-stained MAC681-treated K-562 cells by green-stained macrophages (Fig. 4F).

However, neither morphological assessments of dying cells nor the release of the above-mentioned biochemical correlates, alone or in combination, can predict with complete certainty the induction of bona fide ICD [49]. The gold-standard approach to proving the immunogenicity of the cellular demise induced by a specific agent relies on vaccination experiments in immunocompetent mice. Hence, we treated syngeneic murine myeloid C1498 cancer cells with the MAC681 at different concentrations for 24 h and evaluated cell death induction by flow cytometry after annexin V/PI staining (Supp. Fig. S4D). Next, syngeneic immunocompetent C57BL/6 mice were vaccinated with dying cancer cells treated either with 5 µM MAC681 or a combination of 2.5 µM cytarabine (CTB) and 0.5 µM doxorubicin (DOX), a known induction therapy, into the right flanks. Control mice (non-vaccinated) were given 1 × PBS only. After one week, all mice were challenged with living C1498 cells into the contralateral flank of the mice, and tumor growth was monitored until the tumor volume reached > 600 mm^3^ in the non-vaccinated control group. We showed that tumor volume and weight were significantly reduced in the MAC681-treated group compared to the control group (Fig. 4G). Moreover, the MAC681 effect on tumor growth was similar to the impact of the induction therapy (CTB + DOX). In addition, we assessed the effect of MAC681 vaccination on spleen morphology and revealed that MAC681 significantly reduced spleen weight but did not impact its length (Fig. 4H). Altogether, these data provide strong evidence supporting the immunogenic potential of indolequinone MAC681.

### Synergistic potential of MAC681 in combination with asciminib in imatinib-sensitive and -resistant human leukemia

**Fig. 5:**
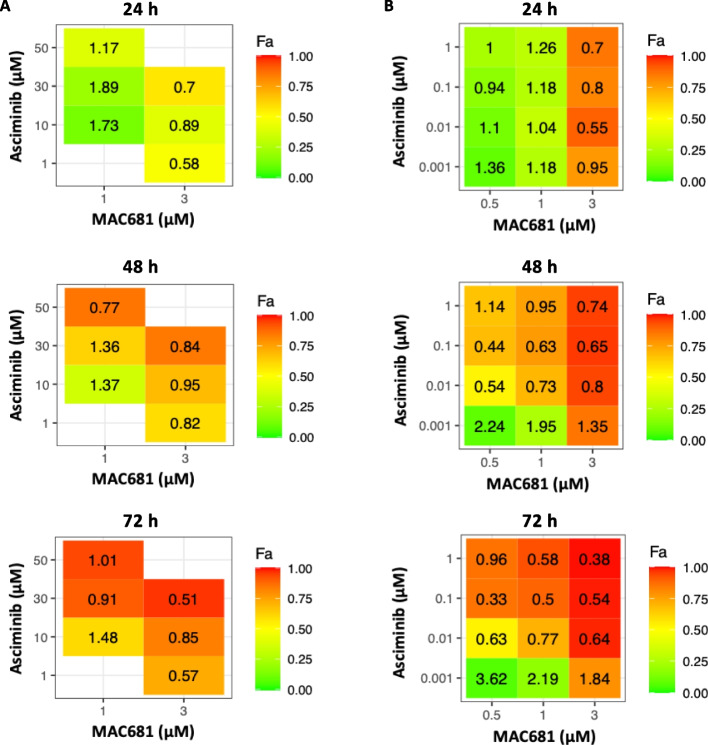
Synergistic effects of MAC681 with asciminib in K-562 and K-562R cells. Heatmap showing the inhibition of cell viability analyzed by trypan blue exclusion test in K-562R [**A**] and K-562 [**B**] cells by different combinatory treatments at 24 h, 48 h, and 72 h. The fraction affected (Fa) scale is shown as a sidebar, where 0 = no inhibitory effect and 1 = 100% inhibition of cell viability. The combinatory index was calculated by Compusyn software and is indicated within each quadrant (CI < 1 synergism, CI = 1 additive effect, CI > 1 antagonism)

Despite improvements in CML therapy by developing efficient TKIs suppressing BCR-ABL activity, one in five patients develops resistance to TKIs. Moreover, 22% of de novo CML- patients in the chronic phase become intolerant (JALSG CML212 study), requiring discontinuation of the therapy because of adverse side effects. Accordingly, combining TKIs with non-ABL-related therapeutic strategies is required to prevent the onset and overcome the appearance of TKI-resistant clones or diminish the side effects of TKIs. Hence, we investigated the effect of the combination of MAC681 with the ATP-non-competitive, allosteric TKI asciminib in multi-TKI-resistant and sensitive K-562 cell models up to 72 h. We used the Chou-Talalay algorithm to determine the combination index and fraction-affected values. Our results in K-562R cells showed a synergistic effect of 3 µM MAC681 with 1, 10, and 30 µM of asciminib at any given time point (Fig. 5A). In K-562 sensitive cells, multiple combinations showed synergistic effects during 24 h, 48 h, and 72 h (Fig. 5B).

## Discussion

CML cells express the BCR-ABL1 fusion oncogene, which leads to profound deregulation of the gene expression and, consequently, protein expression of leukemic cells. These deregulations lead to ROS production, DNA repair pathway deregulation, and a metabolic adaptation conferring proliferation and survival advantage. CML cells are particularly addicted to these forms of metabolic and functional dysregulations. CML cells are in high energy demand, and metabolic perturbations lead to insufficient energy production. Pharmacologic intervention can address this metabolic state characterized by increased glycolytic metabolism. Here, we demonstrate the capacity of the indolequinone MAC681 to induce metabolic catastrophe followed by the induction of non-canonical controlled necrosis to “kill unkillable cells” [50].

Targeting these above-mentioned essential pathways in addition to TKI-mediated interference with BCR-ABL1-driven signaling pathways is considered a promising antileukemic approach. We previously targeted the accumulation of ROS in CML cells with the hydroquinone TMQ0153, leading to the accumulation of intracellular ROS levels followed by ATP loss, autophagy, leading concentration-dependently to apoptosis, and controlled necrosis [51]. Whereas normal HSCs and mature CML cells rely predominantly on glycolysis [6], CML LSCs use oxidative phosphorylation for survival [5]. However, there is conflicting information regarding the energy metabolism of TKI-resistant CML cells. Patel et al. showed that stem cells from non-mutational resistant patients rely on glycolysis due to a progressive failure of the electron transport chain. This metabolic adaptation was attributed to STAT-3-mediated reprogramming of the mitochondrial metabolism [52]. Accordingly, the use of metabolic inhibitors, including the inhibitor of glucose metabolism by 2-Deoxy-d-glucose (2-DG), reduced CML cell viability, and a co-treatment of 2-DG with imatinib led to synergistic autophagic cell death [53]. Interestingly, in response to imatinib, BCR-ABL-driven human leukemia cells significantly reduced glucose consumption and lactate secretion and increased intracellular ATP levels, indicating a switch from glycolysis to mitochondrial oxidative phosphorylation [54, 55].

Hence, unsurprisingly, other groups provided evidence that treatment-resistant CML cells appear to be OXPHOS-dependent [5, 8]. Similar to the latter, comparing the metabolic profile between K-562 sensitive and K-562R cells in our lab, we detected that multi-resistant K-562 cells display a higher OXPHOS metabolism than the parental K-562 (data not shown).

Moreover, patient cells resistant to imatinib presented differential levels of amino acids and acylcarnitines [56], accumulation of citric acid intermediates, and increased levels of NADH [57], altogether witnessing a profound rewiring of the mitochondrial metabolic capacity and underlining the essential role of mitochondrial metabolism in CML resistance. As leukemic cells are addicted to NAD for proliferation and survival, we postulate that new drug entities targeting mitochondrial physiology and reducing NADH levels can act as potent cytotoxic agents against sensitive and resistant CML blasts. Consequently, MAC681 reduced the NADH levels, contributing to reduced ATP production and subsequently controlled necrosis (Fig. 2B-F).

DNA damage repair is a biological process deregulated in CML, providing advantages and disadvantages for CML blasts [58]. Indeed, the rapid proliferation rate of CML cells requires rapid cell cycle progress without extensive pausing in S and early G2 phases due to DNA repair. Accordingly, using rapid alternative nonhomologous end-joining (ALT-NHEJ), CML cells achieve a growth advantage despite the accumulation of mutations, eventually leading to genomic instability and chromosomal condensation [24]. Targeting additional components of the faulty CML DNA repair pathways, for example, by inhibiting the essential DNA repair factor PARP1, could constitute a helpful therapeutic strategy to be exploited by single or combined treatments, especially as PARP1 and DNA ligase IIIα levels were described as biomarkers in resistant CML patients. Targeting the ALT-NHEJ pathway when treatment with TKIs failed was previously targeted by L67, a DNA ligase I and IIIα inhibitor, and the PARP1 inhibitor NU1025 [13]. In opposition to our data showing a complete degradation of PARP1 by MAC681 alone, none of these agents had the potential to affect CML cells. The authors showed, nevertheless, a specific sensitivity of resistant CML cell lines when combined, validating the strategy of targeting the ALT-NHEJ pathway as an interesting therapeutic target, not only in CML but also in other cancers relying on the same mechanisms. Considering that imatinib treatment could reprogram DNA repair in the first days of therapy [59], we postulate that a combined treatment by ALT-NHEJ inhibitors and BCR-ABL1 inhibitors could be particularly efficient and prevent the emergence of resistance at later time points. In that sense, we assessed the effect of combined treatment by the PARP1-inhibiting indolequinone MAC681 and asciminib, belonging to the specifically targeting the ABL myristoyl pocket anti-BCR-ABL1 drug category. So far, few combination treatments against CML involving asciminib have been published. Synergistic interactions of asciminib with imatinib or dasatinib had been observed in KCL-22 CML cells, whereas other authors observed antagonism between asciminib and the other drugs [60]. Interestingly, from an immunologic point of view, T cells treated with asciminib and other TKIs maintained their metabolic plasticity, witnessing metabolic fitness and flexibility. Moreover, asciminib presented a more moderate inhibitory effect of T cell activation, beneficial for controlling CML by immune effectors [61]. Here, we show the first synergetic induction of cell death by MAC681 combined with asciminib, opening new windows for innovative anti-CML treatments.

Resistance to physiological and tolerogenic apoptosis is widely considered a cancer and CML resistance factor. Therefore, searching for pharmacologically active compounds leading to non-canonical forms of cell death is essential. MAC681 belongs to this category of pre-clinically assessed drug candidates. We observed rapid loss of ATP and NADH, accompanied by DNA damage accumulation and progressive degradation of the mitochondrial integrity, leading to cell death that was sensitive to 3-aminobenzamide. The late release of truncated AIF is reminiscent of parthanatos, a form of PARP-1-dependent cell death involving the generation of PAR followed by the nuclear translocation of AIF, resulting in caspase-independent cell death [62]. We were unable to show hyperparylation (data not shown). In contrast, we consider that the late release of AIF from the mitochondria is a consequence of mitochondrial degradation rather than a regulated step of parthanatos.

Targeting the metabolism of myeloid leukemia is a promising therapeutic approach. The small molecule CCI-006 was described as a novel inhibitor of mixed lineage leukemia (MLL)-rearranged, and CALM-AF10 translocated myeloid leukemia cells by targeting a metabolic vulnerability in a subset of low HIF1alpha/low MEIS1-expressing MLL-rearranged leukemia cells that present more glycolytic metabolic phenotype [63].

NQO1 appears to be an essential and central regulator of cellular metabolism, as a perturbation of this enzymatic function by indolequinones may contribute to mitochondrial dysfunction. In opposition to MAC681, the compound KL1333 reacts with NQO1 as a substrate, increasing intracellular NAD^+^ and ATP levels. As a result, this compound decreased lactate and ROS levels and increased mitochondrial mass, membrane potential, and oxidative capacity [64], demonstrating the profound metabolic effect of positive and negative modulation of NQO1. β-Lapachone is bioactivated by NQO1 by a mechanism different from MAC681, as β-lapachone triggers a futile redox cycle that consumes oxygen and generates high levels of ROS. As a result, β-lapachone treatment leads to DNA damage and rapid PARP1-mediated NAD^+^ consumption. These results show that NAD depletion is an essential mechanism to kill leukemia cells with particularly elevated metabolic needs. A combined NAD(P)^+^ depletion and inhibition of glycolysis reduces ATP levels similar to MAC681 treatment. Moore et al. further exacerbated this effect by using the nicotinamide phosphoribosyl transferase (NAMPT) inhibitor FK866, which contributed to an even stronger depletion of NAD^+^ pools as a strategy to target NQO1^+^ cancer types [65]. Chen et coll. described NQO1-mediated necrosis as noptosis in the case of β-lapachone-induced cell death [66]. Considering the essential contribution of ROS to noptosis initiation, we believe that MAC681-induced controlled necrosis differs in part from noptosis, considering the absence of redox cycling. In agreement with Chen et coll., we believe NQO1 remains an essential regulator of non-canonical cell death modalities.

3ABA is the most used first-generation PARP inhibitor. It is known to enhance radiation-induced cell death by sensitizing cancer cells to radiation and, as such, augmenting the antitumor efficacy [67, 68]. Moreover, using 3ABA in combination with DNA-damaging drugs became an attractive strategy against resistant cancer cells to enhance the apoptotic activity of these drugs [69–72]. Despite its non-toxic properties and initial high specificity towards PARP, some off-target effects have been reported in mammalian and plant systems. Several studies demonstrated its PARP-independent mechanisms, including inhibition of microbe-associated molecular patterns (MAMPs)-induced callose deposition in plants [73], indirect inhibition of protein kinase C in U-937 cells [74] or its antioxidative potential as a hydroxyl radical scavenger in the cell-free system of the rat brain cortex [75]. In addition, 3ABA mitigated chemically-induced toxicity by inhibiting a cytochrome P450-dependent metabolic activation of dichlobenil in the mouse olfactory mucosa [76]. However, we present evidence of a novel off-target effect of 3ABA for the first time. Due to its ability to act as a reaction partner (such as a Michael donor), 3ABA could paradoxically function as an antagonist and decrease the efficacy of the anticancer drugs when used in combinatory treatment. In general, many α,β-unsaturated compounds possess cytotoxic properties as DNA might get alkylated by attacking the β carbon of such compounds. Hence, once 3ABA interacts with indolequinone MAC681, it loses its ability to cause detrimental DNA damage. This discovery is important considering that 3ABA is abundantly used as a *bona fide* PARP1 inhibitor, and prior evaluation of the chemical structures of the compounds selected for the combinatory studies with 3ABA is necessary to exclude candidates that would fit Michael acceptor characteristics or other possible reaction-partners.

MAC681 has a dual impact, inducing DNA damage while concurrently blocking its repair. We describe here that the prevention of PARP1 degradation by an inhibitor, which blocks its repair activity, should not block the initiation of DNA damage. Our experimental findings indicate that the protective effect of 3ABA is not solely attributed to preventing the degradation of PARP1 but is likely a consequence of its role in preventing MAC681-induced DNA damage (Fig. 3F).

For this study, we recognize the distinct nature of CML and AML as separate diseases, yet they share a common hematological myeloid differentiation path. The cell lines used represent different stages of this path, and our intention was to demonstrate the broader applicability of MAC681 across the myeloid differentiation pathway. Recent advances in genomics and molecular biology have revealed a complex landscape of genetic and molecular alterations in myeloid leukemia. These findings underscore the heterogeneity of the disease and the fluid nature of its classification as new molecular subtypes continue to be identified. Despite potential mechanistic differences, the MAC681's ability to induce immunogenic cell death and exhibit anti-leukemic effects in both leukemia types highlights its promising therapeutic potential across various myeloid malignancies.

Altogether, we present a pharmacological approach leading to the deletion of PARP1, an essential cofactor for DNA repair. We exploit leukemia-intrinsic DNA damage response defects known to induce immunogenicity phenotypes likely to lead to activation of the immune cycle. Indeed, PARP1 inhibition could lead to immunogenic neoantigen accumulation, presented on the surface of dying CML cells, recognized by the immune system to improve immune recognition of cells otherwise poorly immunogenic. Our results show, in addition, downregulation of the immune proteasome as well as likely reduced antigen presentation capacity of the patient’s CML cells. This effect is further amplified by ROS accumulation and mitochondrial integrity disruption as mitochondrial DAMPs, known to play a nefarious role in chronic inflammation, could contribute to improving immune recognition of dying CML cells by immune effectors. The similarity between mitochondria and bacteria was evoked to understand the activation of innate immune mechanisms [77]. We validated the release of DAMPs and the increased immunogenicity of myeloid leukemia cells by an in vivo vaccination approach, achieving a significantly reduced tumor growth after vaccination with dying myeloid cells. We predict that this type of vaccination could be further improved by using immune checkpoint inhibitors. Altogether, increasing the tumor mutational burden by PARP1 inhibition and mitochondrial deregulation could add CML to the cancer types that could benefit from immunotherapy.

### Supplementary Information

**Additional file 1: Supplementary Figure 1.** [S1] Voronoi diagram of the superpathway and its children, yellow indicates matched entities and gradient represents p-value for [A] GSE5550, [B] Combined dataset.

**Additional file 2: Supplementary Figure 2.** [S2A] MAC681 treatment reduces the basal oxygen consumption rate in K-562 cells (double-sided unpaired t-test **p ≤ 0.01). [S2B] K-562 cells depend more on mitochondrial ATP than glycolytic ATP production. MAC681 treatment specifically reduces the mitochondrial ATP production rate (double-sided unpaired t-test **p ≤ 0.01). [S2C] Mitochondrial Ca^2+^ co-localization was confirmed by the calculation of Manders' coefficients (co-occurrence), Pearson's coefficient (correlation), and Spearman's coefficient (correlation). [S2D] Time-dependent translocation of AIF from mitochondria to the nucleus induced by MAC681 (5 µM). Immunofluorescence microscopy analysis of K-562 cells treated or not with MAC681, incubated with the AIF antibody, and counter-stained with Hoechst. Representative pictures from three independent experiments are shown. [S2E] Time-dependent translocation of AIF from the cytoplasm to the nucleus in K-562 cells, assessed by western blot. Tubulin and lamin B were used as loading controls. After quantifying the bands of interest, cytoplasmic and nuclear proteins were normalized to tubulin and lamin B, respectively (double-sided unpaired t-test *p ≤ 0.05, ****p ≤ 0.0001). Representative western blots from three independent experiments are shown. [S2F] MAC681-induced PARP1 degradation after 4 hours of treatment was prevented by calpain inhibitor PD 150606 (1-hour pre-treatment) in K-562 cells, assessed by western blot. β-Actin was used as a loading control. A representative picture from three independent experiments is shown. [S2G] Effect of MAC681 on leukemia cell line viability. The IC_50_ value was defined as the compound concentration needed to inhibit 50 % cell viability compared to untreated controls. [S2H] Differential toxicity of MAC681 in PBMCs and K-562 cells (left panel) or RPMI1788 and K-562 cells (right panel) was estimated based on results with trypan blue exclusion assay (One-way ANOVA, Šídák's multiple comparisons test ***p ≤ 0.001, ****p ≤ 0.0001). [S2I] Colony formation assay with K-562 cells treated with MAC681 at the indicated concentrations. Quantification of total MTT signal (One-way ANOVA, Dunnett’s multiple comparisons test ***p ≤ 0.001, ****p ≤ 0.0001). [S2J] Colony formation assay with U-937, Meg-01, KBM-5, and HL-60 cells treated with MAC681 at the indicated concentrations. Images are representative of three independent experiments. Neither viability nor proliferation of the leukemia cells was affected by MAC681 before seeding the pretreated cells on methylcellulose (right panels) (One-way ANOVA, Dunnett’s multiple comparisons test *p ≤ 0.05, **p ≤ 0.01, ***p ≤ 0.001, ****p ≤ 0.0001). [S2K] Effect of MAC681 on K-562 (upper panel) and U-937 (lower panel) tumor formation in zebrafish xenograft assays. Representative images from 10 fish per condition. PBS injection served as a negative control. Error bars represent the SD of at least three independent experiments. The fluorescent U-937 tumor mass was quantified and reported in the graph (One-way ANOVA, Dunnett’s multiple comparisons test *p ≤ 0.05, **p ≤ 0.01, ***p ≤ 0.001, ****p ≤ 0.0001).

**Additional file 3: Supplementary Figure 3.** [S3A] Kinetic analysis of the PARP1 protein levels in K-562 cells treated with MAC681 at 5 µM. β-actin was used as a loading control. Etoposide (VP16) and ethanol 10% (Et10%) were used as positive controls. [S3B] RT-PCR analysis of PARP1 mRNA expression at indicated time points. K-562 cells were treated with MAC681 (5 µM), 3ABA (5 mM), or their combination (One-way ANOVA, Šídák's multiple comparisons test *p ≤ 0.05, **p ≤ 0.01). [S3C] Kinetic analysis of the PARP1 protein levels in K-562 cells treated with MAC681 at 5 µM with or without cycloheximide (CHX). β-actin was used as a loading control. Mcl-1 protein expression with short turnover was used as a positive control for CHX activity. [S3D] The control docking experiment of PARP1 with 2, 3-dihydrobenzofuran-7-carboximade derivative (2US). The 2US in the complex structure of PARP1 (PDB ID: 4OQA) and the docking model are shown as red and blue stick models, respectively. Oxygen atoms are visualized in orange (upper panel). MAC681 has a similar binding mode to 2US in a complex structure of PARP1 (PDB ID: 4OQA) and PJ34 in a complex structure of PARP1 (PDB ID: 4UXB). MAC681, 2US, and PJ34 are represented as stick models in magenta, red, and yellow, respectively (lower panel). PatchDock server and AutoDock4 program were used in our docking simulation studies. [S3E] The formation of γH2AX nuclear foci in U-937, MOLM-13, MV4-11, and OCI-AML3 was quantified by flow cytometry after 4 h of MAC681 treatment (left panel) (One-way ANOVA, Dunnett’s multiple comparisons test **p ≤ 0.01, ***p ≤ 0.001, ****p ≤ 0.0001). Nuclear morphology was assessed by fluorescent microscopy with Hoechst staining at the same time point (right panel). [S3F] Percentage of cell death was quantified with Hoechst/PI staining in U-937 cells treated either by MAC681 (5 µM), 3ABA (1mM), VP16 (100 µM) or in a combination of 3ABA with MAC681 or with VP16 at 24 hours (upper panel). Western blot analysis of PARP1 protein cleavage in U-937 cells was determined simultaneously (lower panel). Etoposide (VP16) served as a positive control for apoptosis induction and PARP1 cleavage. [S3G] 3ABA-MAC681 adduct formation in K-652 cells (duplicate experiment). The 3ABA-MAC681-6-position adduct was detected by mass spectrometry in K-652 cells. Experimental conditions are described in the Materials and Methods section, *Mass Spectrometry*. MAC681-alcohol and trifluorophenol (TFP) are metabolites of MAC681. [S3H] Effect of washout of 3ABA pre-treatment on the protective effect against MAC681-mediated early and late cellular responses. Intracellular ATP content in K-562 cells pre-treated with 3ABA (5 mM) for 30 min, with or without washout, and treated for 4 hours with MAC681 (5µM) normalized to control cells was measured by CellTiter-Glo (upper left panel). Number of viable cells (upper middle panel) and viability (upper right panel) were assessed by trypan blue exclusion test after 24 h (two-way ANOVA, Fisher’s test *p ≤ 0.05, **p ≤ 0.01, ***p ≤ 0.001, ****p ≤ 0.0001). The nuclear morphology of cells was visualized using Hoechst/PI staining by fluorescent microscopy (lower panel) at 24 h. [S3I] Kinetic analysis of reactive oxygen species (ROS) levels measured by flow cytometry after MAC681 (5 µM) treatment with dichlorofluorescein diacetate (DCFDA) staining (upper panel) or with redox sensor red (RSR) staining (lower panel). H_2_O_2_ was used as a positive control for ROS induction (double-sided unpaired t-test *p ≤ 0.05, ****p ≤ 0.0001). [S3J] 2 e^-^ reductase-dependent bioreductive mechanism of MAC681 activation *via* the iminium salt. [S3K] 2 e^-^ reductase-dependent bioreductive mechanism of MAC681 activation *via* the quinone methide. [S3L] NQO1 (upper panel) and NQO2 (lower panel) expression levels in CML patient stem cells compared to stem cells of healthy volunteers in indicated datasets (GSE5550, GSE97562+ GSE47927) (double-sided unpaired t-test *p ≤ 0.05, **p ≤ 0.01, ***p ≤ 0.001). [S3M] NQO1 (upper panel) and NQO2 (lower panel) gene expression profiles in different CML cancer cell lines from the CCLE dataset.

**Additional file 4: Supplementary Figure 4.** [S4A] Dose-dependent increase in IL-8 mRNA expression induced by MAC681 at 8 h analyzed by RT PCR (One-way ANOVA, Dunnett’s multiple comparisons test **p ≤ 0.01). [S4B] Time-dependent release of HMGB1 after treatment with 5 µM of MAC681 assessed by ELISA in OCI-AML3 cells supernatants (double-sided unpaired t-test *p ≤ 0.05, **p ≤ 0.01, ***p ≤ 0.001, ****p ≤ 0.0001). [S4C] Dose-dependent HMGB1 release after treatment with indicated concentrations of MAC681 assessed by ELISA in C1498 cells supernatants after 24 hours (One-way ANOVA, Dunnett’s multiple comparisons test **p ≤ 0.01, ****p ≤ 0.0001). [S4D] Quantification of cell death induced after 24 hours by MAC681 at indicated concentrations in C1498 cells analyzed by Annexin V/PI staining (One-way ANOVA, Dunnett’s multiple comparisons test ***p ≤ 0.001, ****p ≤ 0.0001). [S4E] Kinetic analysis of the weights of C57BL/6 mice (One-way ANOVA, Dunnett’s multiple comparisons test).

**Additional file 5.**

**Additional file 6.**

## Data Availability

No datasets were generated or analysed during the current study.

## Abbreviations

3ABA: 3-Aminobenzamide
ABL: Abelson
AIF: Apoptosis-inducing factor
AML: Acute myeloid leukemia
ALT-NHEJ: Alternative nonhomologous DNA end joining
ATCC: American type culture collection
BCR: Breakpoint cluster region
BRCA: Breast cancer gene
CCCP: Carbonyl cyanide 3-chlorophenylhydrazone
CCLE: Cancer Cell Line Encyclopedia
CHX: Cycloheximide
CI: Combination index
CML: Chronic myeloid leukemia
cNHEJ: Classic nonhomologous DNA end joining
CTB: Cytarabine
DAMPs: Damage-associated molecular patterns
2-DG: 2-Deoxy-d-glucose
DEGs: Differentially expressed genes
DMSO: Dimethyl sulfoxide
DNA-PK: DNA-dependent protein kinase
DOX: Doxorubicin
DSMZ: Deutsche Sammlung für Mikroorganismen und Zellkulturen
FAO: Fatty acid oxidation
FBS: Fetal bovine serum
FC: Fold change
FCS: Fetal calf serum
FDR: False discovery rate
FITC: Fluorescein isothiocyanate
GO: Gene ontology
GSE: Gene set enrichment
GSEA: GSE analysis
HMGB1: High mobility group box 1
HR: Homologous recombination
HSC: Hematopoietic stem cell
ICD: Immunogenic cell death
IL: Interleukin
K-562R: Imatinib-resistant K-562
LSC: Leukemia stem cell
Mcl-1: Myeloid cell leukemia 1
MFI: Median fluorescent intensity
MLL: Mixed lineage leukemia
MPER: Mammalian protein extraction reagent
NAD: Nicotinamide adenine dinucleotide
NAM: Nicotinamide
NES: Normalized enrichment score
NQO: NAD(P)H quinone dehydrogenase
OCR: Oxygen consumption rate
OXPHOS: Oxidative phosphorylation
PARP: Poly(ADP-ribose) polymerase
PBMC: Peripheral blood mononuclear cell
PBS: Phosphate-buffered saline
PDB: Protein data bank
PMSF: Phenylmethylsulfonyl fluoride
PI: Propidium iodide
qPCR: Quantitative PCR
RT-PCR: Reverse transcription polymerase chain reaction
ROS: Reactive oxygen species
SDS: Sodium dodecyl sulfate
SERCA: Sarcoplasmic/endoplasmic reticulum Ca^2+^-ATPase
siRNA: Small interfering RNA
SIRT: Sirtuin
TCA: Tricarboxylic acid cycle
TKI: Tyrosine kinase inhibitor
TMB: Tetramethylbenzidine
TSG: Thapsigargin
VP-16: Etoposide

## References

[1] Cortes JE, Jones D, O'Brien S, Jabbour E, Ravandi F, Koller C (2010). Results of dasatinib therapy in patients with early chronic-phase chronic myeloid leukemia. J Clin Oncol.

[2] Kantarjian HM, Shah NP, Cortes JE, Baccarani M, Agarwal MB, Undurraga MS (2012). Dasatinib or imatinib in newly diagnosed chronic-phase chronic myeloid leukemia: 2-year follow-up from a randomized phase 3 trial (DASISION). Blood.

[3] Zulbaran-Rojas A, Lin HK, Shi Q, Williams LA, George B, Garcia-Manero G (2018). A prospective analysis of symptom burden for patients with chronic myeloid leukemia in chronic phase treated with frontline second- and third-generation tyrosine kinase inhibitors. Cancer Med.

[4] Rossari F, Minutolo F, Orciuolo E (2018). Past, present, and future of Bcr-Abl inhibitors: from chemical development to clinical efficacy. J Hematol Oncol.

[5] Kuntz EM, Baquero P, Michie AM, Dunn K, Tardito S, Holyoake TL (2017). Targeting mitochondrial oxidative phosphorylation eradicates therapy-resistant chronic myeloid leukemia stem cells. Nat Med.

[6] Simsek T, Kocabas F, Zheng J, Deberardinis RJ, Mahmoud AI, Olson EN (2010). The distinct metabolic profile of hematopoietic stem cells reflects their location in a hypoxic niche. Cell Stem Cell.

[7] Mojtahedi H, Yazdanpanah N, Rezaei N (2021). Chronic myeloid leukemia stem cells: targeting therapeutic implications. Stem Cell Res Ther.

[8] de Beauchamp L, Himonas E, Helgason GV (2022). Mitochondrial metabolism as a potential therapeutic target in myeloid leukaemia. Leukemia.

[9] Liu L, Su X, Quinn WJ, Hui S, Krukenberg K, Frederick DW (2018). Quantitative analysis of NAD synthesis-breakdown fluxes. Cell Metab.

[10] Xie N, Zhang L, Gao W, Huang C, Huber PE, Zhou X (2020). NAD(+) metabolism: pathophysiologic mechanisms and therapeutic potential. Signal Transduct Target Ther.

[11] Muvarak N, Nagaria P, Rassool FV (2012). Genomic instability in chronic myeloid leukemia: targets for therapy?. Curr Hematol Malig Rep.

[12] Sallmyr A, Tomkinson AE, Rassool FV (2008). Up-regulation of WRN and DNA ligase IIIalpha in chronic myeloid leukemia: consequences for the repair of DNA double-strand breaks. Blood.

[13] Tobin LA, Robert C, Rapoport AP, Gojo I, Baer MR, Tomkinson AE, Rassool FV (2013). Targeting abnormal DNA double-strand break repair in tyrosine kinase inhibitor-resistant chronic myeloid leukemias. Oncogene.

[14] Bolton-Gillespie E, Schemionek M, Klein HU, Flis S, Hoser G, Lange T (2013). Genomic instability may originate from imatinib-refractory chronic myeloid leukemia stem cells. Blood.

[15] Ross D, Siegel D, Beall H, Prakash AS, Mulcahy RT, Gibson NW (1993). DT-diaphorase in activation and detoxification of quinones. Bioreductive activation of mitomycin C. Cancer Metastasis Rev.

[16] Reigan P, Colucci MA, Siegel D, Chilloux A, Moody CJ, Ross D (2007). Development of indolequinone mechanism-based inhibitors of NAD(P)H:quinone oxidoreductase 1 (NQO1): NQO1 inhibition and growth inhibitory activity in human pancreatic MIA PaCa-2 cancer cells. Biochemistry.

[17] Colucci MA, Reigan P, Siegel D, Chilloux A, Ross D, Moody CJ (2007). Synthesis and evaluation of 3-aryloxymethyl-1,2-dimethylindole-4,7-diones as mechanism-based inhibitors of NAD(P)H:quinone oxidoreductase 1 (NQO1) activity. J Med Chem.

[18] Qin R, You FM, Zhao Q, Xie X, Peng C, Zhan G, Han B (2022). Naturally derived indole alkaloids targeting regulated cell death (RCD) for cancer therapy: from molecular mechanisms to potential therapeutic targets. J Hematol Oncol.

[19] Krysko O, Aaes TL, Kagan VE, D'Herde K, Bachert C, Leybaert L (2017). Necroptotic cell death in anti-cancer therapy. Immunol Rev.

[20] Hernandez AP, Juanes-Velasco P, Landeira-Vinuela A, Bareke H, Montalvillo E, Gongora R, Fuentes M (2021). Restoring the immunity in the tumor microenvironment: insights into immunogenic cell death in onco-therapies. Cancers (Basel).

[21] Galluzzi L, Vacchelli E, Bravo-San Pedro JM, Buque A, Senovilla L, Baracco EE (2014). Classification of current anticancer immunotherapies. Oncotarget.

[22] Diaz-Blanco E, Bruns I, Neumann F, Fischer JC, Graef T, Rosskopf M (2007). Molecular signature of CD34(+) hematopoietic stem and progenitor cells of patients with CML in chronic phase. Leukemia.

[23] Gautier L, Cope L, Bolstad BM, Irizarry RA (2004). affy–analysis of Affymetrix GeneChip data at the probe level. Bioinformatics.

[24] Aviles-Vazquez S, Chavez-Gonzalez A, Hidalgo-Miranda A, Moreno-Lorenzana D, Arriaga-Pizano L, Sandoval-Esquivel MA (2017). Global gene expression profiles of hematopoietic stem and progenitor cells from patients with chronic myeloid leukemia: the effect of in vitro culture with or without imatinib. Cancer Med.

[25] Abraham SA, Hopcroft LE, Carrick E, Drotar ME, Dunn K, Williamson AJ (2016). Dual targeting of p53 and c-MYC selectively eliminates leukaemic stem cells. Nature.

[26] Parker HS, Corrada Bravo H, Leek JT (2014). Removing batch effects for prediction problems with frozen surrogate variable analysis. PeerJ.

[27] Kohlmann A, Kipps TJ, Rassenti LZ, Downing JR, Shurtleff SA, Mills KI (2008). An international standardization programme towards the application of gene expression profiling in routine leukaemia diagnostics: the microarray Innovations in LEukemia study prephase. Br J Haematol.

[28] Ghandi M, Huang FW, Jane-Valbuena J, Kryukov GV, Lo CC, McDonald ER (2019). Next-generation characterization of the cancer cell line encyclopedia. Nature.

[29] Ritchie ME, Phipson B, Wu D, Hu Y, Law CW, Shi W, Smyth GK (2015). limma powers differential expression analyses for RNA-sequencing and microarray studies. Nucleic Acids Res.

[30] Wickham H, Wickham H (2016). Build a Plot Layer by Layer. ggplot2.

[31] Gillespie M, Jassal B, Stephan R, Milacic M, Rothfels K, Senff-Ribeiro A (2022). The reactome pathway knowledgebase 2022. Nucleic Acids Res.

[32] Orlikova B, Tasdemir D, Golais F, Dicato M, Diederich M (2011). The aromatic ketone 4'-hydroxychalcone inhibits TNFalpha-induced NF-kappaB activation via proteasome inhibition. Biochem Pharmacol.

[33] Schneider CA, Rasband WS, Eliceiri KW (2012). NIH Image to ImageJ: 25 years of image analysis. Nat Methods.

[34] Yan JS, Yang MY, Zhang XH, Luo CH, Du CK, Jiang Y (2022). Mitochondrial oxidative phosphorylation is dispensable for survival of CD34(+) chronic myeloid leukemia stem and progenitor cells. Cell Death Dis.

[35] Pujana MA, Han JD, Starita LM, Stevens KN, Tewari M, Ahn JS (2007). Network modeling links breast cancer susceptibility and centrosome dysfunction. Nat Genet.

[36] O'Sullivan CC, Moon DH, Kohn EC, Lee JM (2014). Beyond breast and ovarian cancers: PARP Inhibitors for BRCA mutation-associated and BRCA-like solid tumors. Front Oncol.

[37] Paulet L, Trecourt A, Leary A, Peron J, Descotes F, Devouassoux-Shisheboran M (2022). Cracking the homologous recombination deficiency code: how to identify responders to PARP inhibitors. Eur J Cancer.

[38] Kamel D, Gray C, Walia JS, Kumar V (2018). PARP inhibitor drugs in the treatment of breast, ovarian, prostate and pancreatic cancers: an update of clinical trials. Curr Drug Targets.

[39] listed Na (2022). AZD5305 more tolerable than earlier PARP Agents. Cancer Discov.

[40] Bai P, Nagy L, Fodor T, Liaudet L, Pacher P (2015). Poly(ADP-ribose) polymerases as modulators of mitochondrial activity. Trends Endocrinol Metab.

[41] Fouquerel E, Sobol RW (2014). ARTD1 (PARP1) activation and NAD(+) in DNA repair and cell death. DNA Repair (Amst).

[42] Rasola A, Bernardi P (2011). Mitochondrial permeability transition in Ca(2+)-dependent apoptosis and necrosis. Cell Calcium.

[43] Vosler PS, Sun D, Wang S, Gao Y, Kintner DB, Signore AP (2009). Calcium dysregulation induces apoptosis-inducing factor release: cross-talk between PARP-1- and calpain-signaling pathways. Exp Neurol.

[44] Lernoux M, Schnekenburger M, Losson H, Vermeulen K, Hahn H, Gerard D (2020). Novel HDAC inhibitor MAKV-8 and imatinib synergistically kill chronic myeloid leukemia cells via inhibition of BCR-ABL/MYC-signaling: effect on imatinib resistance and stem cells. Clin Epigenetics.

[45] Losson H, Gajulapalli SR, Lernoux M, Lee JY, Mazumder A, Gerard D (2020). The HDAC6 inhibitor 7b induces BCR-ABL ubiquitination and downregulation and synergizes with imatinib to trigger apoptosis in chronic myeloid leukemia. Pharmacol Res.

[46] Yan C, Shieh B, Reigan P, Zhang Z, Colucci MA, Chilloux A (2009). Potent activity of indolequinones against human pancreatic cancer: identification of thioredoxin reductase as a potential target. Mol Pharmacol.

[47] He M, Sheldon PJ, Sherman DH (2001). Characterization of a quinone reductase activity for the mitomycin C binding protein (MRD): Functional switching from a drug-activating enzyme to a drug-binding protein. Proc Natl Acad Sci U S A.

[48] Sukkurwala AQ, Martins I, Wang Y, Schlemmer F, Ruckenstuhl C, Durchschlag M (2014). Immunogenic calreticulin exposure occurs through a phylogenetically conserved stress pathway involving the chemokine CXCL8. Cell Death Differ.

[49] Kepp O, Senovilla L, Kroemer G (2014). Immunogenic cell death inducers as anticancer agents. Oncotarget.

[50] Jin S, DiPaola RS, Mathew R, White E (2007). Metabolic catastrophe as a means to cancer cell death. J Cell Sci.

[51] Song S, Lee JY, Ermolenko L, Mazumder A, Ji S, Ryu H (2020). Tetrahydrobenzimidazole TMQ0153 triggers apoptosis, autophagy and necroptosis crosstalk in chronic myeloid leukemia. Cell Death Dis.

[52] Patel SB, Nemkov T, Stefanoni D, Benavides GA, Bassal MA, Crown BL (2021). Metabolic alterations mediated by STAT3 promotes drug persistence in CML. Leukemia.

[53] Li Y, Zeng P, Xiao J, Huang P, Liu P (2022). Modulation of energy metabolism to overcome drug resistance in chronic myeloid leukemia cells through induction of autophagy. Cell Death Discov.

[54] Gottschalk S, Anderson N, Hainz C, Eckhardt SG, Serkova NJ (2004). Imatinib (STI571)-mediated changes in glucose metabolism in human leukemia BCR-ABL-positive cells. Clin Cancer Res.

[55] De Rosa V, Monti M, Terlizzi C, Fonti R, Del Vecchio S, Iommelli F (2019). Coordinate modulation of glycolytic enzymes and OXPHOS by Imatinib in BCR-ABL driven chronic myelogenous leukemia cells. Int J Mol Sci.

[56] Karlikova R, Siroka J, Friedecky D, Faber E, Hrda M, Micova K (2016). Metabolite profiling of the plasma and leukocytes of chronic myeloid leukemia patients. J Proteome Res.

[57] Kluza J, Jendoubi M, Ballot C, Dammak A, Jonneaux A, Idziorek T (2011). Exploiting mitochondrial dysfunction for effective elimination of imatinib-resistant leukemic cells. PLoS ONE.

[58] Pawlowska E, Blasiak J (2015). DNA repair–a double-edged sword in the genomic stability of cancer cells-the case of chronic myeloid leukemia. Int J Mol Sci.

[59] Benito R, Lumbreras E, Abaigar M, Gutierrez NC, Delgado M, Robledo C (2012). Imatinib therapy of chronic myeloid leukemia restores the expression levels of key genes for DNA damage and cell-cycle progression. Pharmacogenet Genomics.

[60] G. Lindström HJ, Friedman R (2020). The effects of combination treatments on drug resistance in chronic myeloid leukaemia: an evaluation of the tyrosine kinase inhibitors axitinib and asciminib. BMC Cancer.

[61] Haselbarth L, Karow A, Mentz K, Bottcher M, Roche-Lancaster O, Krumbholz M (2023). Effects of the STAMP-inhibitor asciminib on T cell activation and metabolic fitness compared to tyrosine kinase inhibition by imatinib, dasatinib, and nilotinib. Cancer Immunol Immunother.

[62] Andrabi SA, Dawson TM, Dawson VL (2008). Mitochondrial and nuclear cross talk in cell death: parthanatos. Ann N Y Acad Sci.

[63] Somers K, Wen VW, Middlemiss SMC, Osborne B, Forgham H, Jung M (2019). A novel small molecule that kills a subset of MLL-rearranged leukemia cells by inducing mitochondrial dysfunction. Oncogene.

[64] Seo KS, Kim JH, Min KN, Moon JA, Roh TC, Lee MJ (2018). KL1333, a Novel NAD(+) modulator, improves energy metabolism and mitochondrial dysfunction in MELAS fibroblasts. Front Neurol.

[65] Moore Z, Chakrabarti G, Luo X, Ali A, Hu Z, Fattah FJ (2015). NAMPT inhibition sensitizes pancreatic adenocarcinoma cells to tumor-selective, PAR-independent metabolic catastrophe and cell death induced by beta-lapachone. Cell Death Dis.

[66] Zhong B, Yu J, Hou Y, Ai N, Ge W, Lu JJ, Chen X (2021). A novel strategy for glioblastoma treatment by induction of noptosis, an NQO1-dependent necrosis. Free Radic Biol Med.

[67] Kelland LR, Tonkin KS (1989). The effect of 3-aminobenzamide in the radiation response of three human cervix carcinoma xenografts. Radiother Oncol.

[68] Dubner D, del Rosario PM, Michelin S, Bourguignon M, Moreau P, Carosella ED, Gisone P (2004). Pharmacological inhibition of DNA repair enzymes differentially modulates telomerase activity and apoptosis in two human leukaemia cell lines. Int J Radiat Biol.

[69] Wang H, Lu C, Li Q, Xie J, Chen T, Tan Y (2014). The role of Kif4A in doxorubicin-induced apoptosis in breast cancer cells. Mol Cells.

[70] Wang H, Lu C, Tan Y, Xie J, Jiang J (2014). Effect of adriamycin on BRCA1 and PARP-1 expression in MCF-7 breast cancer cells. Int J Clin Exp Pathol.

[71] Nguewa PA, Fuertes MA, Cepeda V, Alonso C, Quevedo C, Soto M, Perez JM (2006). Poly(ADP-ribose) polymerase-1 inhibitor 3-aminobenzamide enhances apoptosis induction by platinum complexes in cisplatin-resistant tumor cells. Med Chem.

[72] Jacob DA, Bahra M, Langrehr JM, Boas-Knoop S, Stefaniak R, Davis J (2007). Combination therapy of poly (ADP-ribose) polymerase inhibitor 3-aminobenzamide and gemcitabine shows strong antitumor activity in pancreatic cancer cells. J Gastroenterol Hepatol.

[73] Keppler BD, Song J, Nyman J, Voigt CA, Bent AF (2018). 3-Aminobenzamide Blocks MAMP-induced callose deposition independently of its poly(ADPribosyl)ation inhibiting activity. Front Plant Sci.

[74] Ricciarelli R, Palomba L, Cantoni O, Azzi A (1998). 3-Aminobenzamide inhibition of protein kinase C at a cellular level. FEBS Lett.

[75] Czapski GA, Cakala M, Kopczuk D, Strosznajder JB (2004). Effect of poly(ADP-ribose) polymerase inhibitors on oxidative stress evoked hydroxyl radical level and macromolecules oxidation in cell free system of rat brain cortex. Neurosci Lett.

[76] Eriksson C, Busk L, Brittebo EB (1996). 3-Aminobenzamide: effects on cytochrome P450-dependent metabolism of chemicals and on the toxicity of dichlobenil in the olfactory mucosa. Toxicol Appl Pharmacol.

[77] Dela Cruz CS, Kang MJ (2018). Mitochondrial dysfunction and damage associated molecular patterns (DAMPs) in chronic inflammatory diseases. Mitochondrion.

