# Supplementary figures and images for: Antileukemic potential of methylated indolequinone MAC681 through immunogenic necroptosis and PARP1 degradation
Source: Biomark Res. 2024 May 4;12:47. doi: 10.1186/s40364-024-00594-w (PMC11069214; doi:10.1186/s40364-024-00594-w)

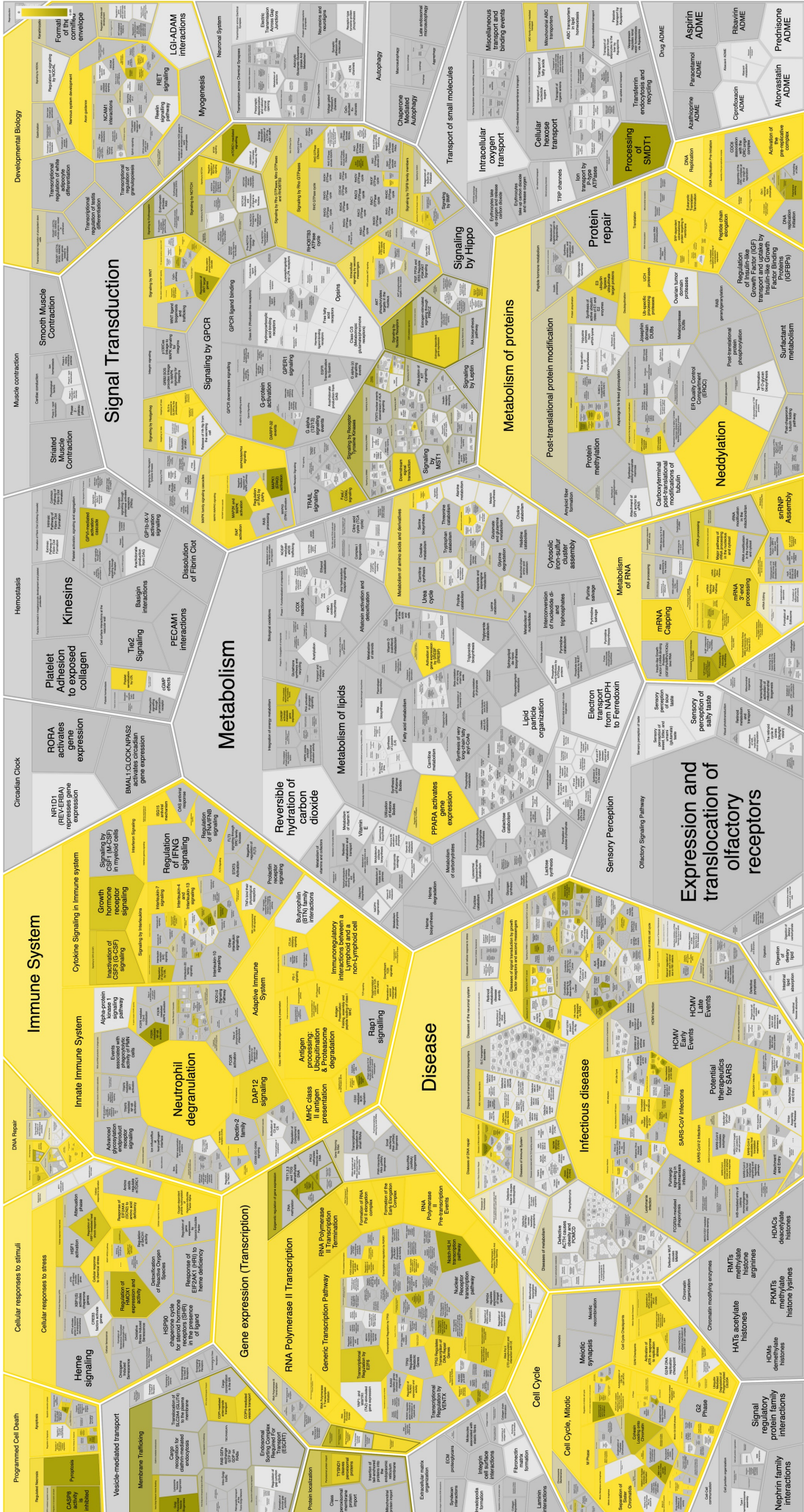

Supplement: Supplementary file 1 — Additional file 1: Supplementary Figure 1. [S1] Voronoi diagram of the superpathway and its children, yellow indicates matched entities and gradient represents p-value for [A] GSE5550, [B] Combined dataset. [file 40364_2024_594_MOESM1_ESM.zip › Boyer_Suppl.Figure1A.pdf]

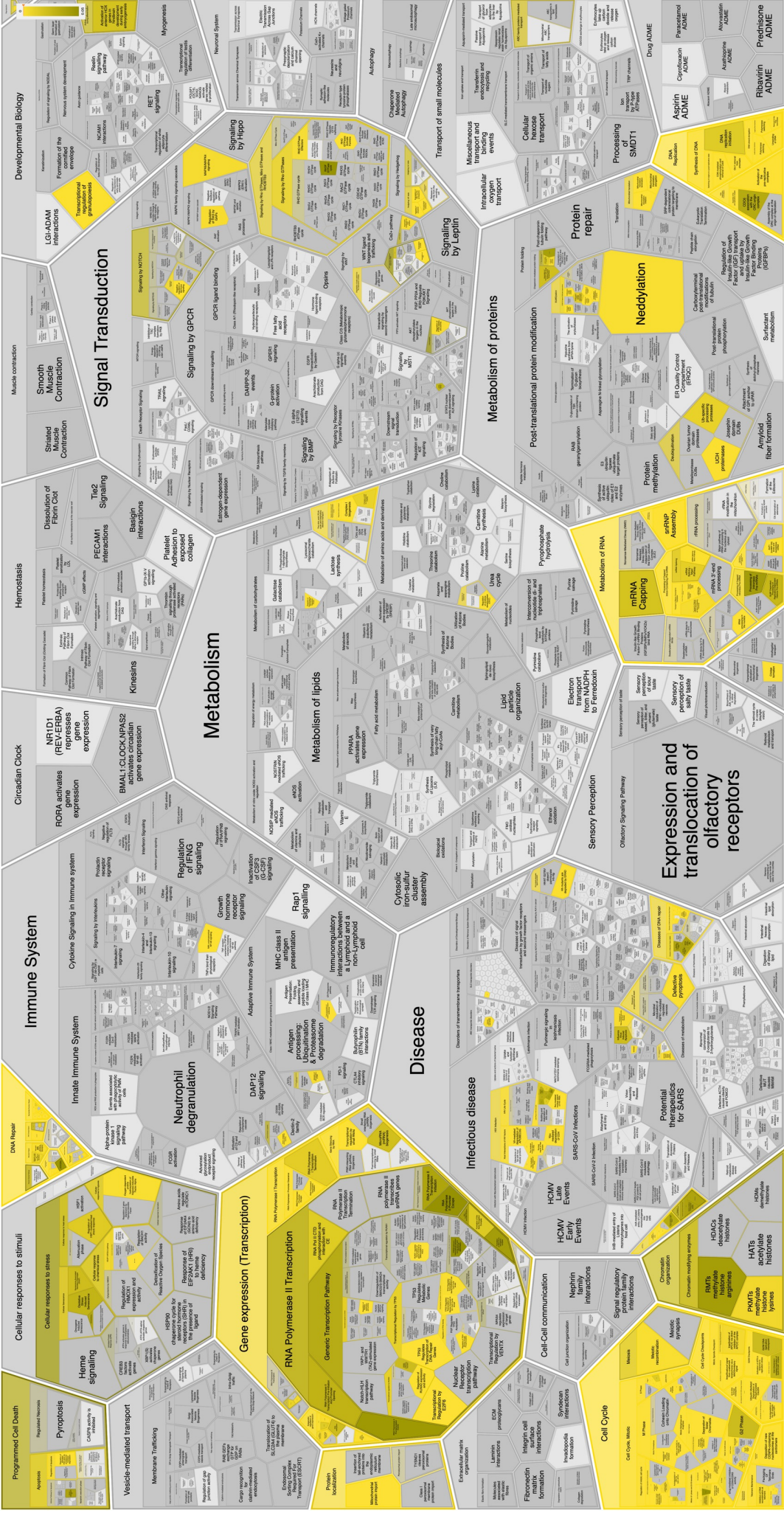

Supplement: Supplementary file 1 — Additional file 1: Supplementary Figure 1. [S1] Voronoi diagram of the superpathway and its children, yellow indicates matched entities and gradient represents p-value for [A] GSE5550, [B] Combined dataset. [file 40364_2024_594_MOESM1_ESM.zip › Boyer_Suppl.Figure1B.pdf]

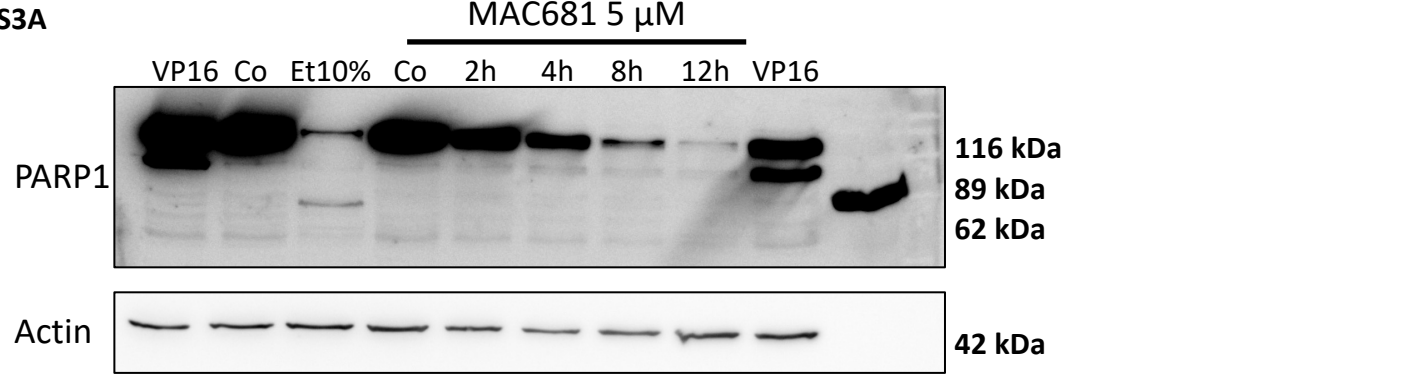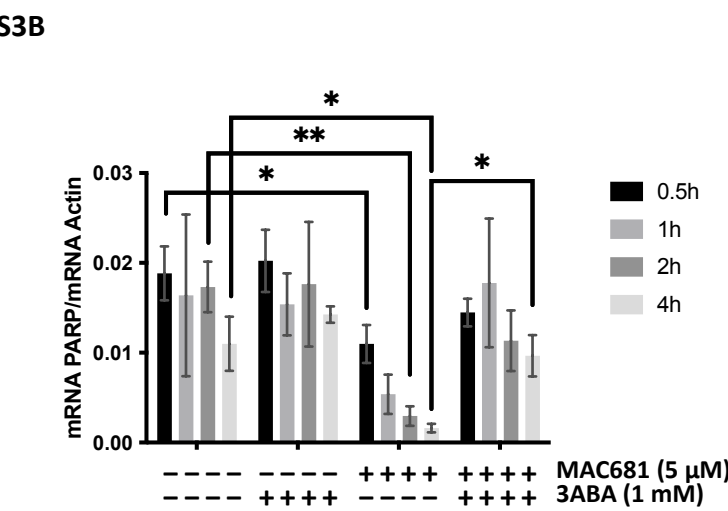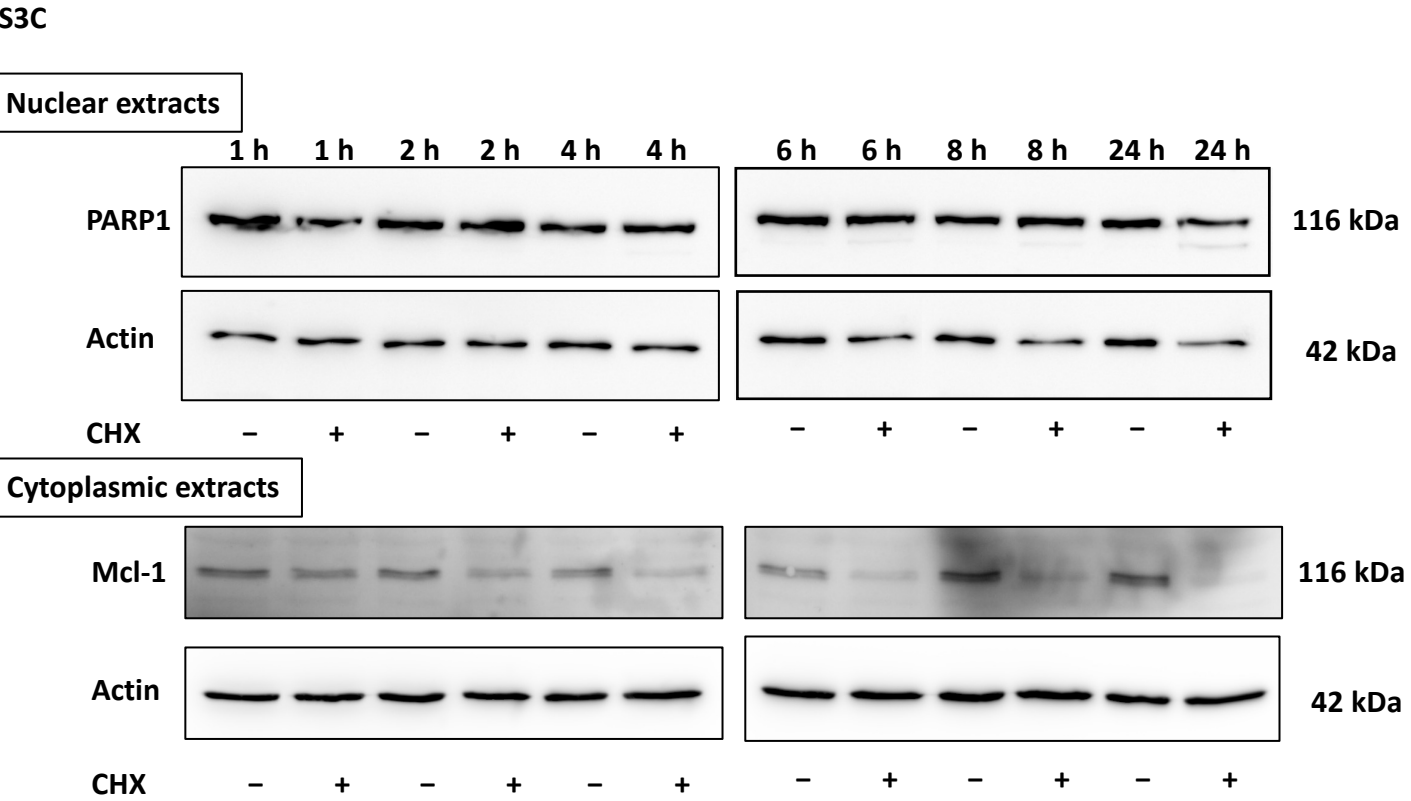

Supplementary figure

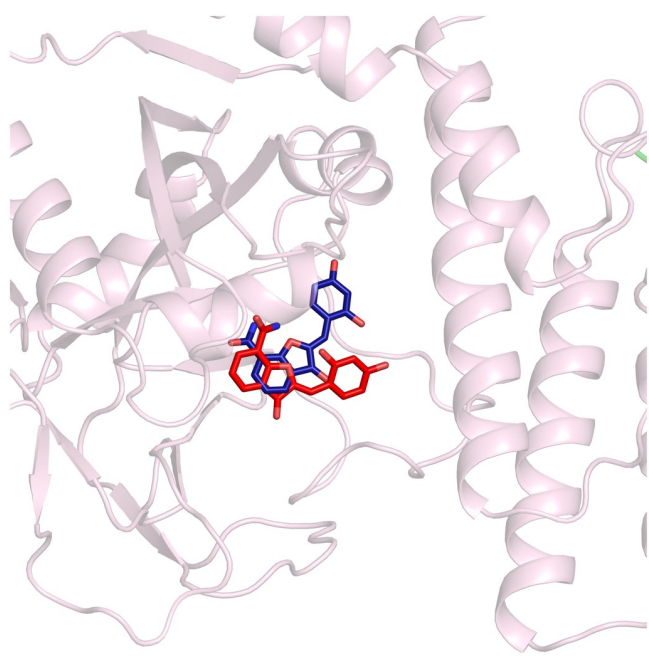

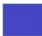 Complex structure

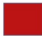 Docking model

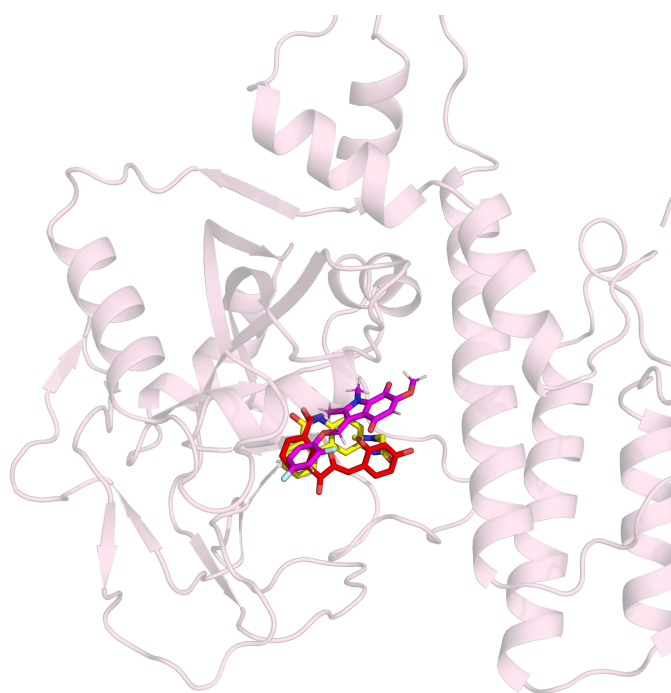

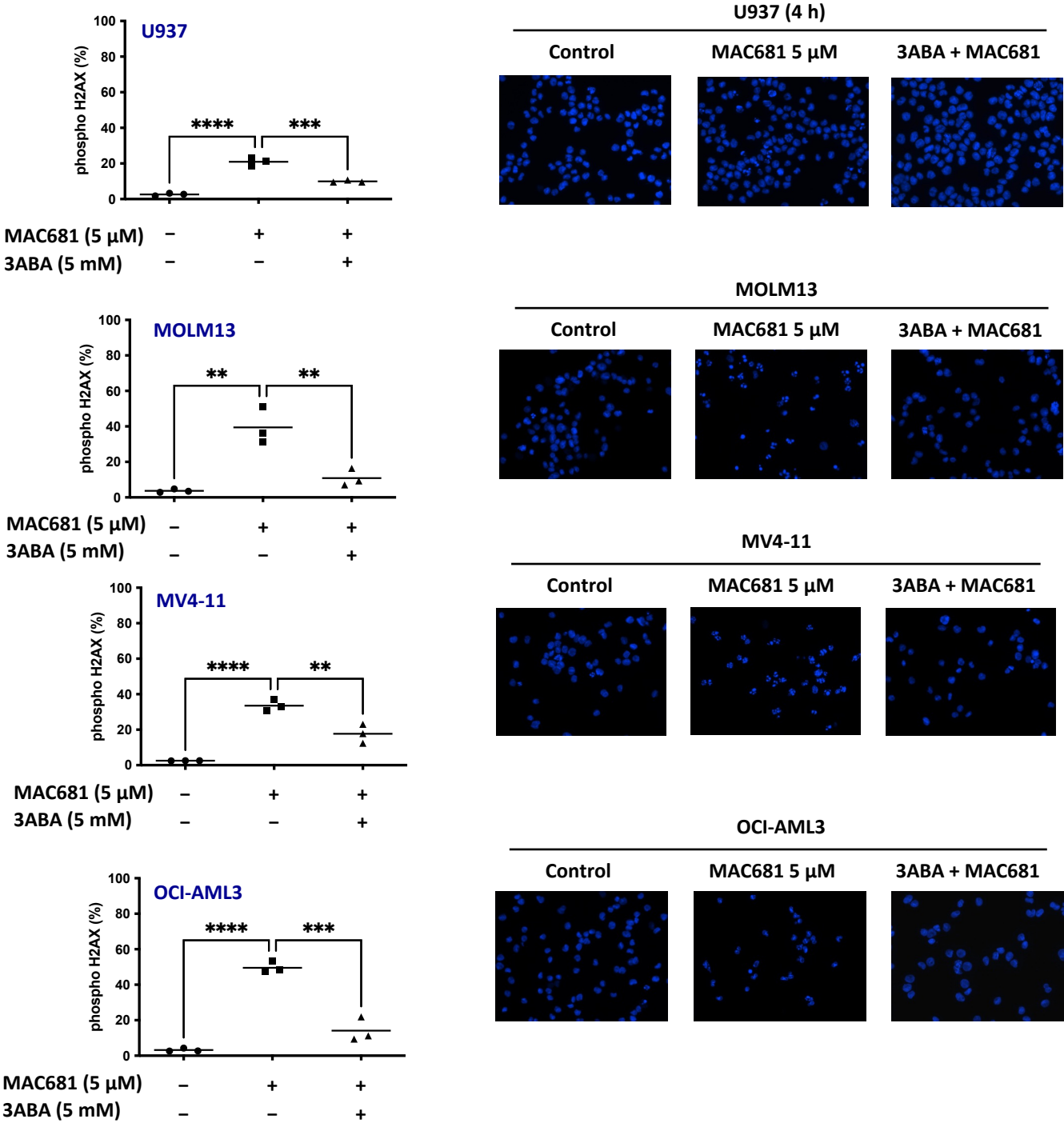

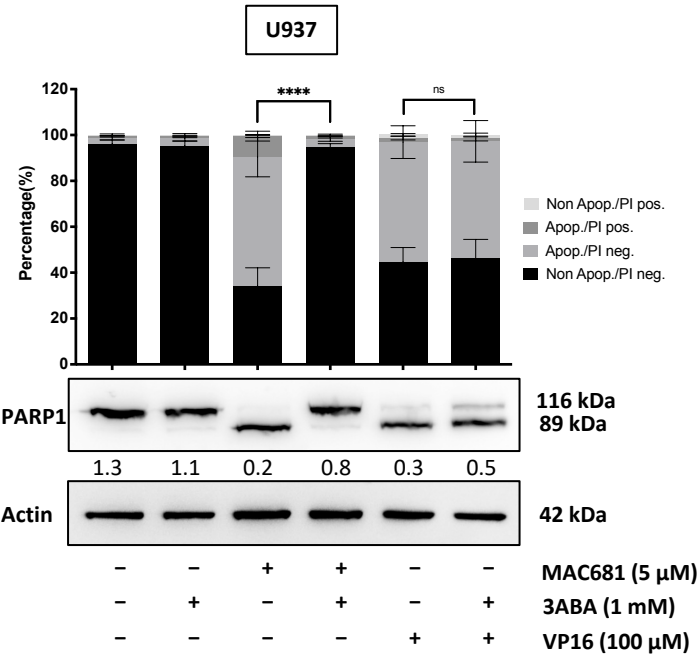

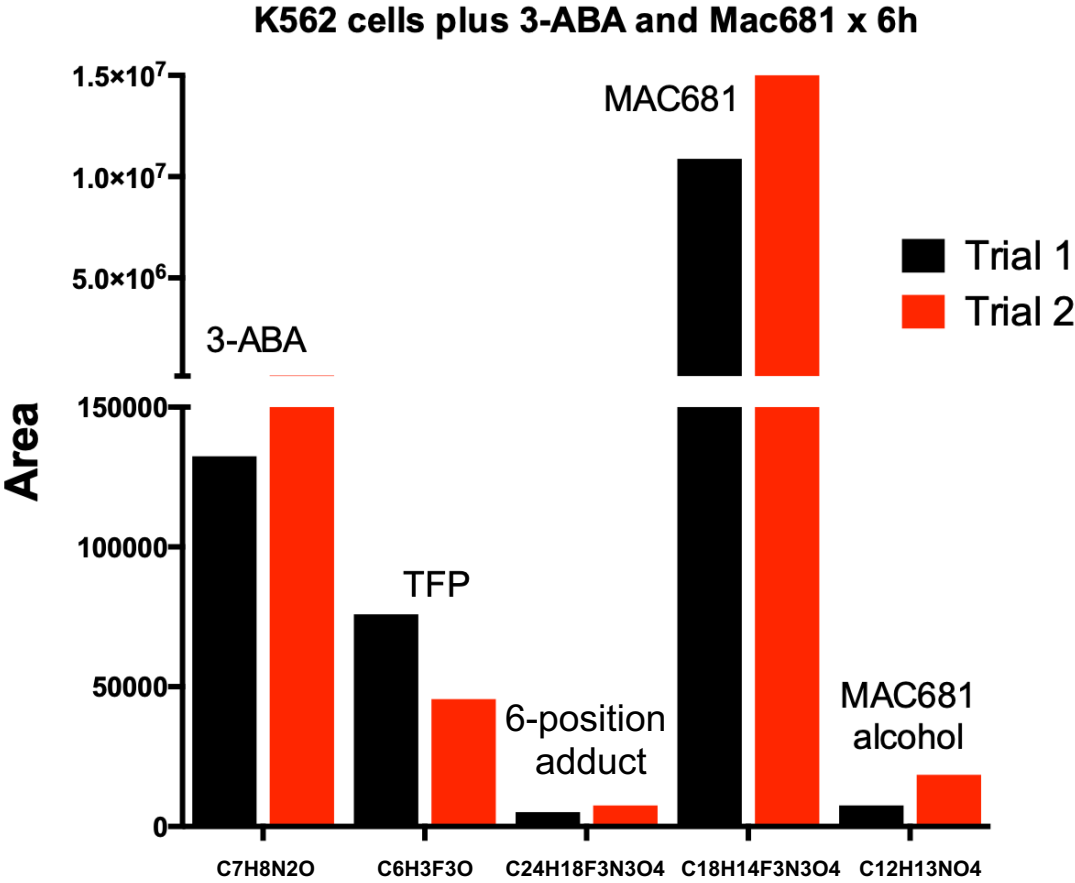

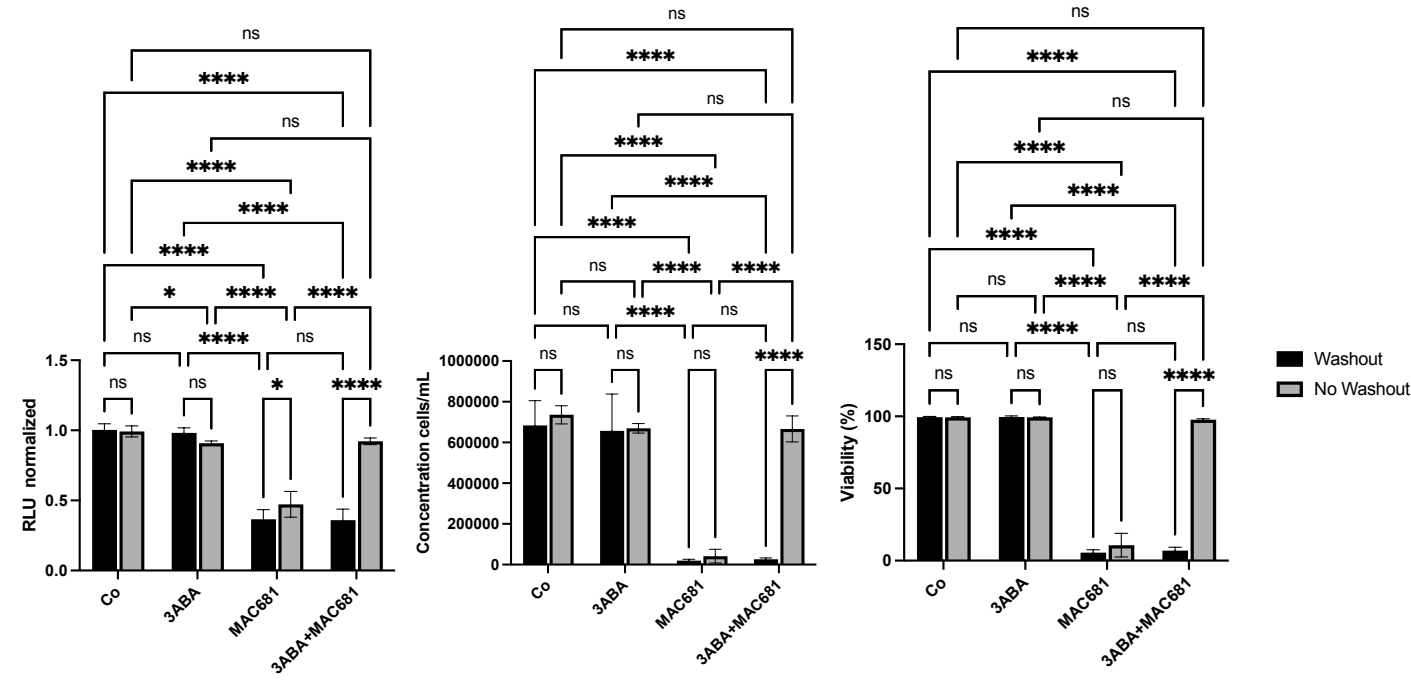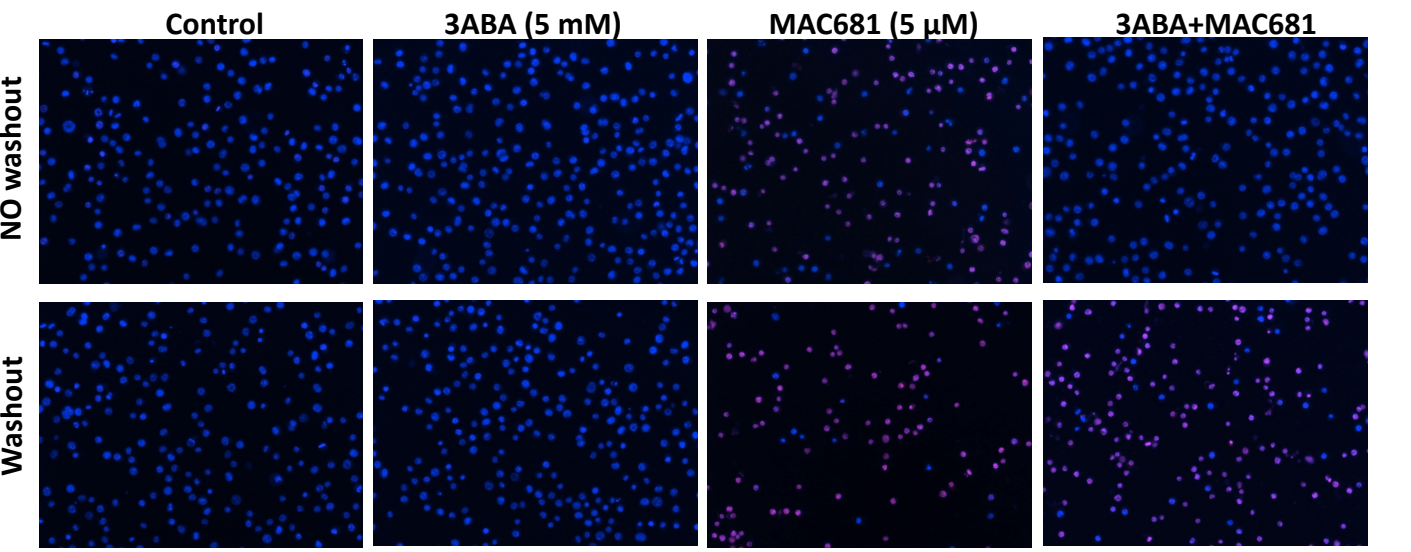

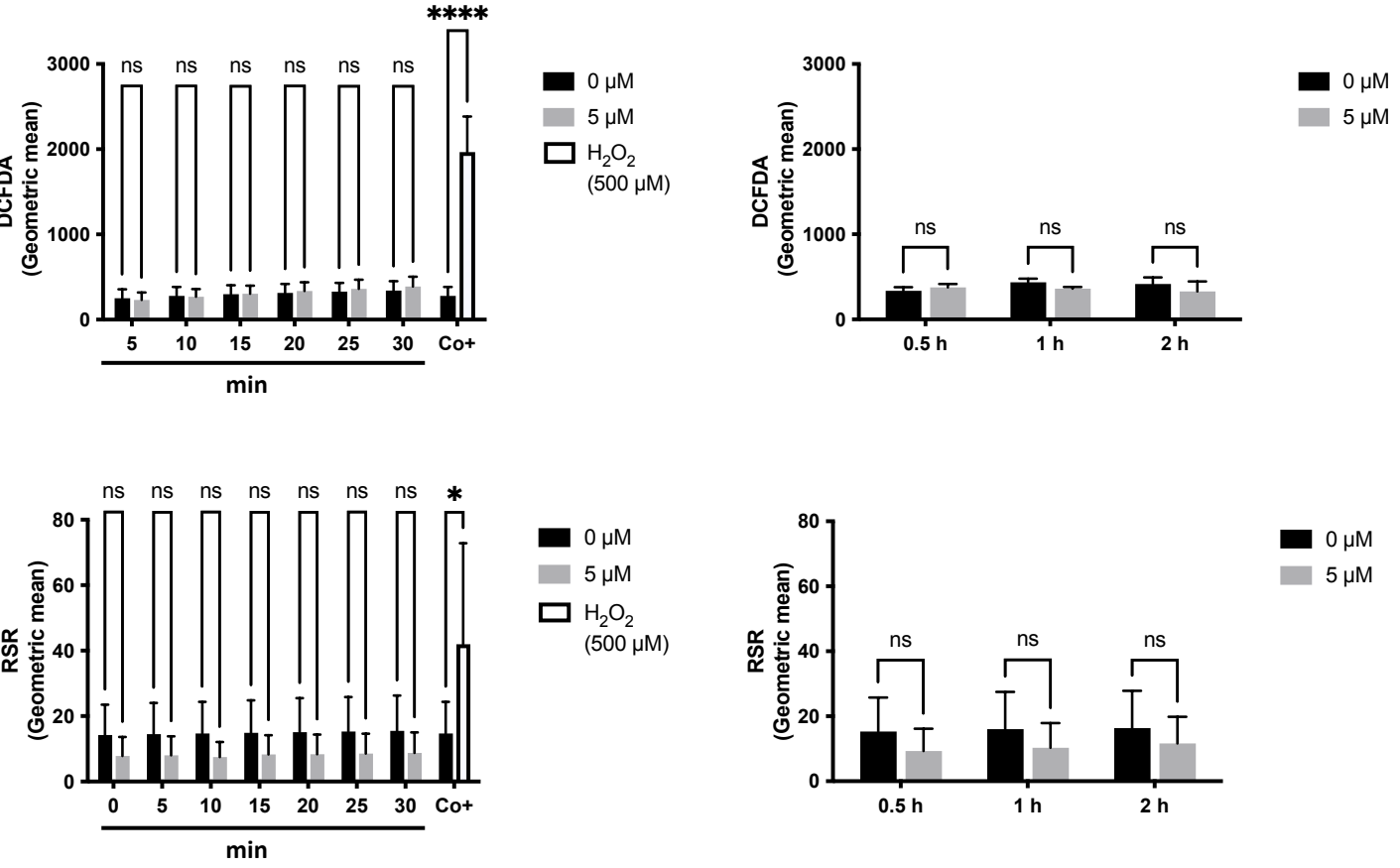

**S3J**

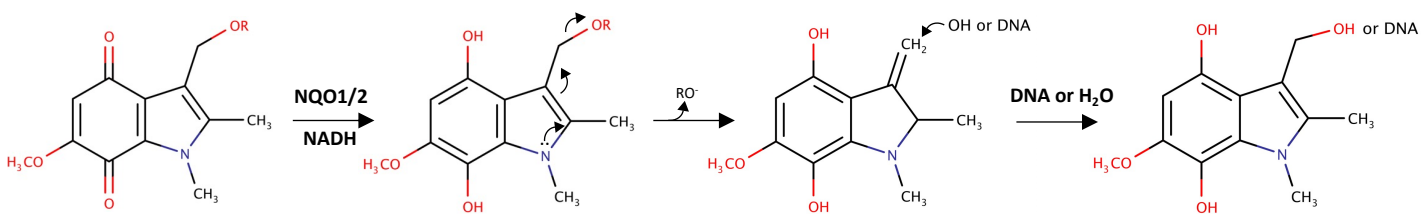

**S3K**

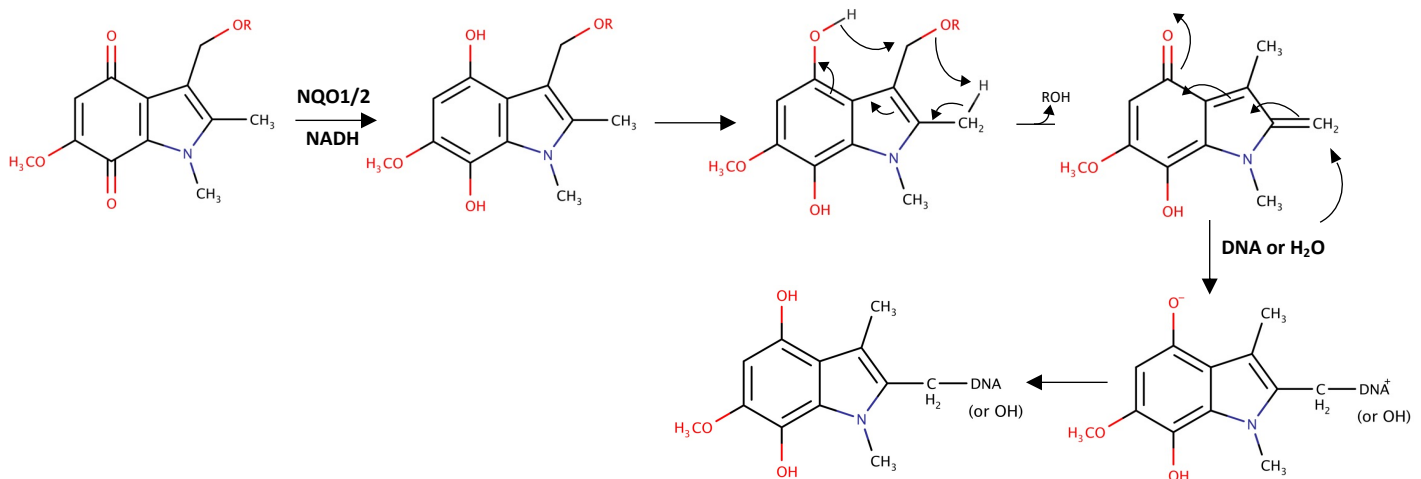

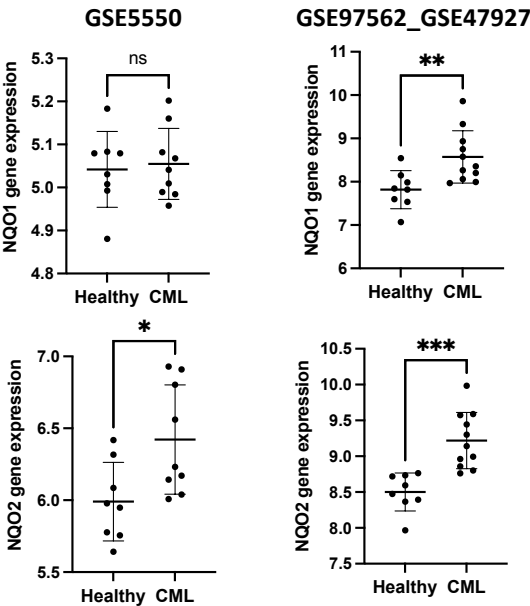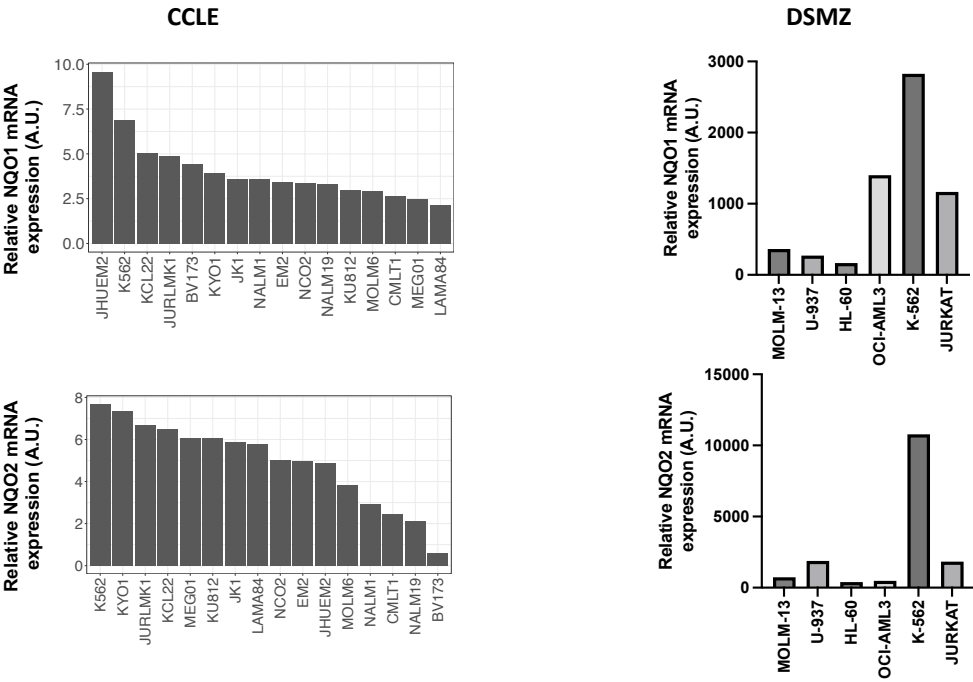

Supplement: Supplementary file 3 — Additional file 3: Supplementary Figure 3. [S3A] Kinetic analysis of the PARP1 protein levels in K-562 cells treated with MAC681 at 5 µM. β-actin was used as a loading control. Etoposide (VP16) and ethanol 10% (Et10%) were used as positive controls. [S3B] RT-PCR analysis of PARP1 mRNA expression at indicated time points. K-562 cells were treated with MAC681 (5 µM), 3ABA (5 mM), or their combination (One-way ANOVA, Šídák's multiple comparisons test *p ≤ 0.05, **p ≤ 0.01). [S3C] Kinetic analysis of the PARP1 protein levels in K-562 cells treated with MAC681 at 5 µM with or without cycloheximide (CHX). β-actin was used as a loading control. Mcl-1 protein expression with short turnover was used as a positive control for CHX activity. [S3D] The control docking experiment of PARP1 with 2, 3-dihydrobenzofuran-7-carboximade derivative (2US). The 2US in the complex structure of PARP1 (PDB ID: 4OQA) and the docking model are shown as red and blue stick models, respectively. Oxygen atoms are visualized in orange (upper panel). MAC681 has a similar binding mode to 2US in a complex structure of PARP1 (PDB ID: 4OQA) and PJ34 in a complex structure of PARP1 (PDB ID: 4UXB). MAC681, 2US, and PJ34 are represented as stick models in magenta, red, and yellow, respectively (lower panel). PatchDock server and AutoDock4 program were used in our docking simulation studies. [S3E] The formation of γH2AX nuclear foci in U-937, MOLM-13, MV4-11, and OCI-AML3 was quantified by flow cytometry after 4 h of MAC681 treatment (left panel) (One-way ANOVA, Dunnett’s multiple comparisons test **p ≤ 0.01, ***p ≤ 0.001, ****p ≤ 0.0001). Nuclear morphology was assessed by fluorescent microscopy with Hoechst staining at the same time point (right panel). [S3F] Percentage of cell death was quantified with Hoechst/PI staining in U-937 cells treated either by MAC681 (5 µM), 3ABA (1mM), VP16 (100 µM) or in a combination of 3ABA with MAC681 or with VP16 at 24 hours (upper panel). Western blot analysis of PARP1 p [file 40364_2024_594_MOESM3_ESM.pdf]

S4A

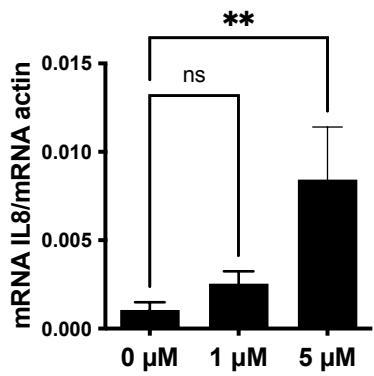

S4B

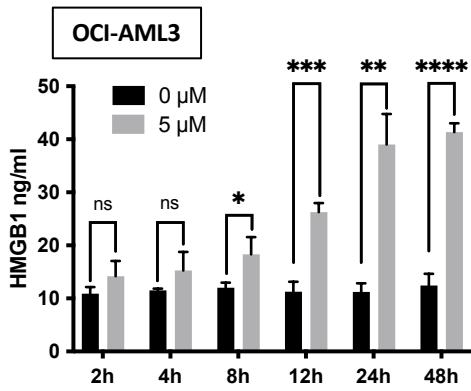

S4C

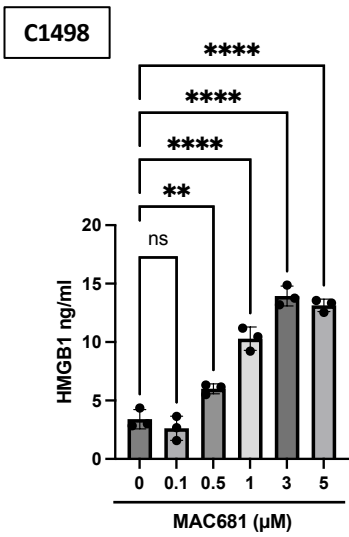

S4D

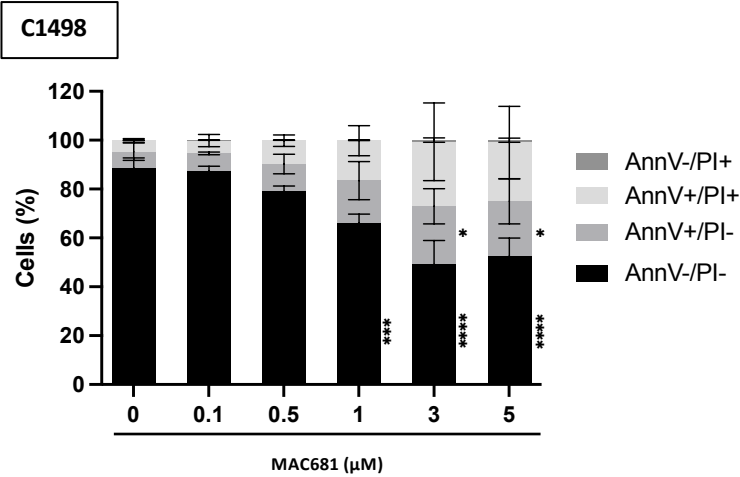

S4E

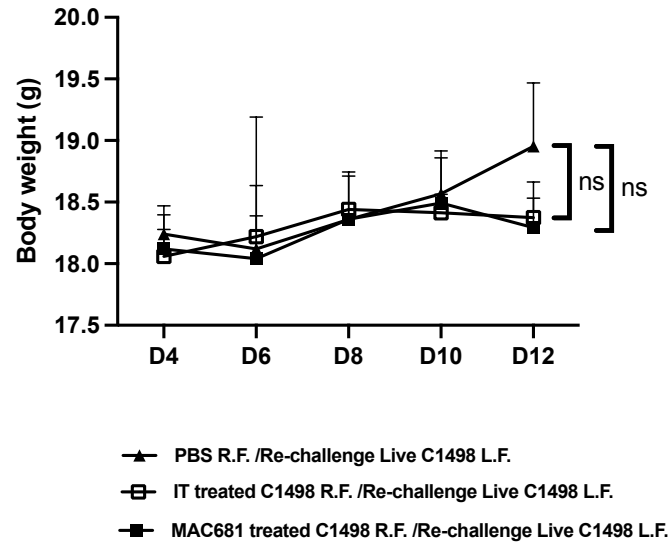

Supplement: Supplementary file 4 — Additional file 4: Supplementary Figure 4. [S4A] Dose-dependent increase in IL-8 mRNA expression induced by MAC681 at 8 h analyzed by RT PCR (One-way ANOVA, Dunnett’s multiple comparisons test **p ≤ 0.01). [S4B] Time-dependent release of HMGB1 after treatment with 5 µM of MAC681 assessed by ELISA in OCI-AML3 cells supernatants (double-sided unpaired t-test *p ≤ 0.05, **p ≤ 0.01, ***p ≤ 0.001, ****p ≤ 0.0001). [S4C] Dose-dependent HMGB1 release after treatment with indicated concentrations of MAC681 assessed by ELISA in C1498 cells supernatants after 24 hours (One-way ANOVA, Dunnett’s multiple comparisons test **p ≤ 0.01, ****p ≤ 0.0001). [S4D] Quantification of cell death induced after 24 hours by MAC681 at indicated concentrations in C1498 cells analyzed by Annexin V/PI staining (One-way ANOVA, Dunnett’s multiple comparisons test ***p ≤ 0.001, ****p ≤ 0.0001). [S4E] Kinetic analysis of the weights of C57BL/6 mice (One-way ANOVA, Dunnett’s multiple comparisons test). [file 40364_2024_594_MOESM4_ESM.pdf]
