# Supplementary material for: Antileukemic potential of methylated indolequinone MAC681 through immunogenic necroptosis and PARP1 degradation
Source: Biomark Res. 2024 May 4;12:47. doi: 10.1186/s40364-024-00594-w (PMC11069214; doi:10.1186/s40364-024-00594-w)
Supplement: Supplementary file 2 — Additional file 2: Supplementary Figure 2. [S2A] MAC681 treatment reduces the basal oxygen consumption rate in K-562 cells (double-sided unpaired t-test **p ≤ 0.01). [S2B] K-562 cells depend more on mitochondrial ATP than glycolytic ATP production. MAC681 treatment specifically reduces the mitochondrial ATP production rate (double-sided unpaired t-test **p ≤ 0.01). [S2C] Mitochondrial Ca2+ co-localization was confirmed by the calculation of Manders' coefficients (co-occurrence), Pearson's coefficient (correlation), and Spearman's coefficient (correlation). [S2D] Time-dependent translocation of AIF from mitochondria to the nucleus induced by MAC681 (5 µM). Immunofluorescence microscopy analysis of K-562 cells treated or not with MAC681, incubated with the AIF antibody, and counter-stained with Hoechst. Representative pictures from three independent experiments are shown. [S2E] Time-dependent translocation of AIF from the cytoplasm to the nucleus in K-562 cells, assessed by western blot. Tubulin and lamin B were used as loading controls. After quantifying the bands of interest, cytoplasmic and nuclear proteins were normalized to tubulin and lamin B, respectively (double-sided unpaired t-test *p ≤ 0.05, ****p ≤ 0.0001). Representative western blots from three independent experiments are shown. [S2F] MAC681-induced PARP1 degradation after 4 hours of treatment was prevented by calpain inhibitor PD 150606 (1-hour pre-treatment) in K-562 cells, assessed by western blot. β-Actin was used as a loading control. A representative picture from three independent experiments is shown. [S2G] Effect of MAC681 on leukemia cell line viability. The IC50 value was defined as the compound concentration needed to inhibit 50 % cell viability compared to untreated controls. [S2H] Differential toxicity of MAC681 in PBMCs and K-562 cells (left panel) or RPMI1788 and K-562 cells (right panel) was estimated based on results with trypan blue exclusion assay (One-way ANOVA, Šídák's multiple compa [file 40364_2024_594_MOESM2_ESM.pdf]

S2A

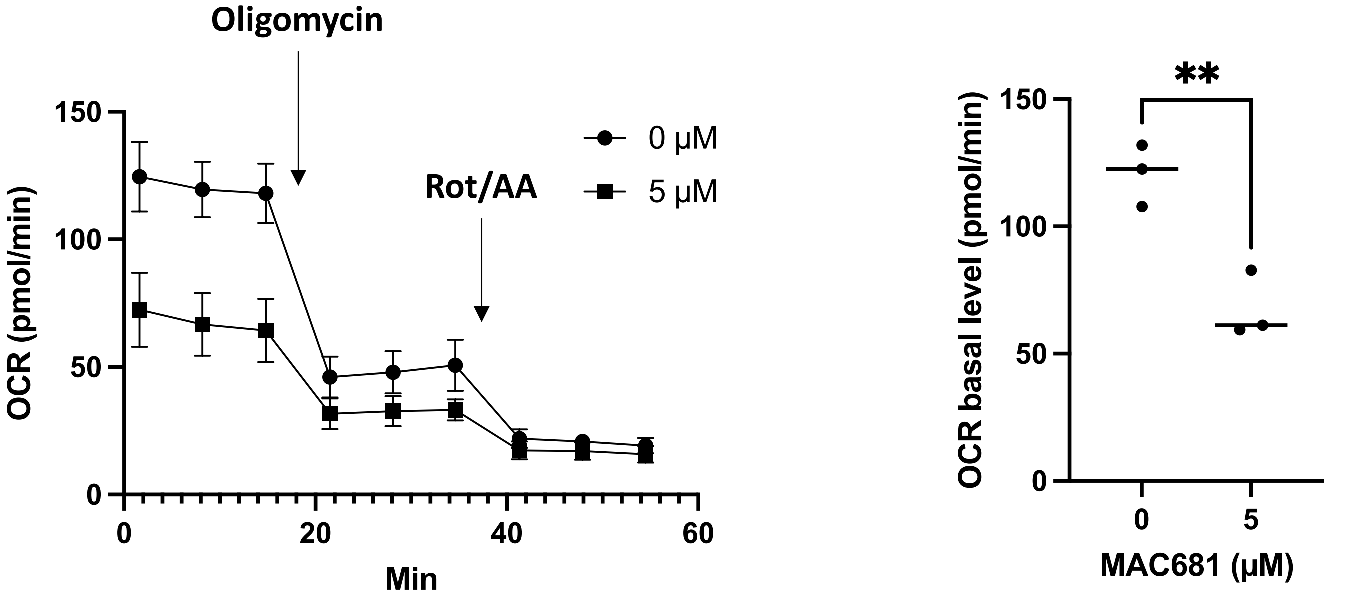

S2B

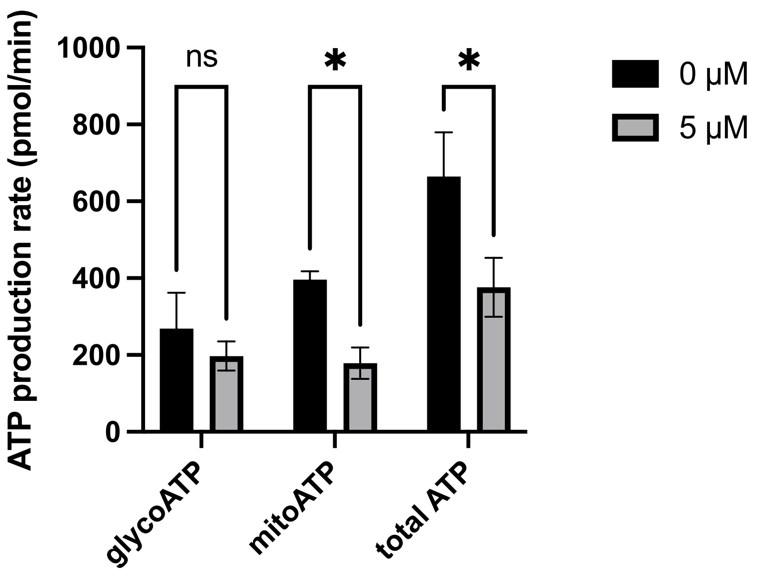

Supplementary figure

S2C

	**Control**	**MAC681**
**Pearson's R**	0.09 ± 0.10	0.40 ± 0.11
**Spearman’s R**	0.08 ± 0.05	0.41 ± 0.11
**Manders' tM1**	0.23 ± 0.18	0.52 ± 0.06
**Manders’ tM2**	0.13 ± 0.10	0.47 ± 0.15

S2D

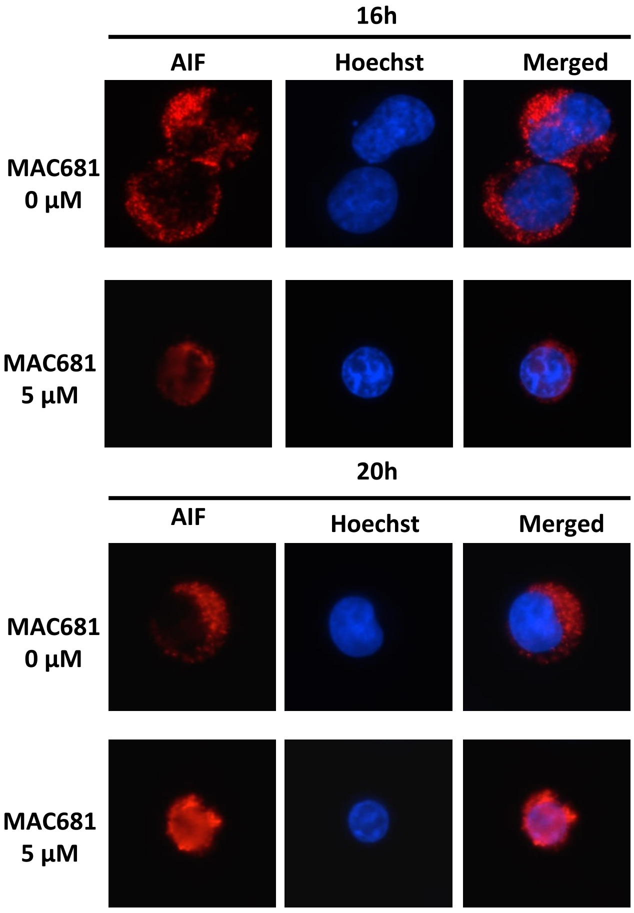

S2E

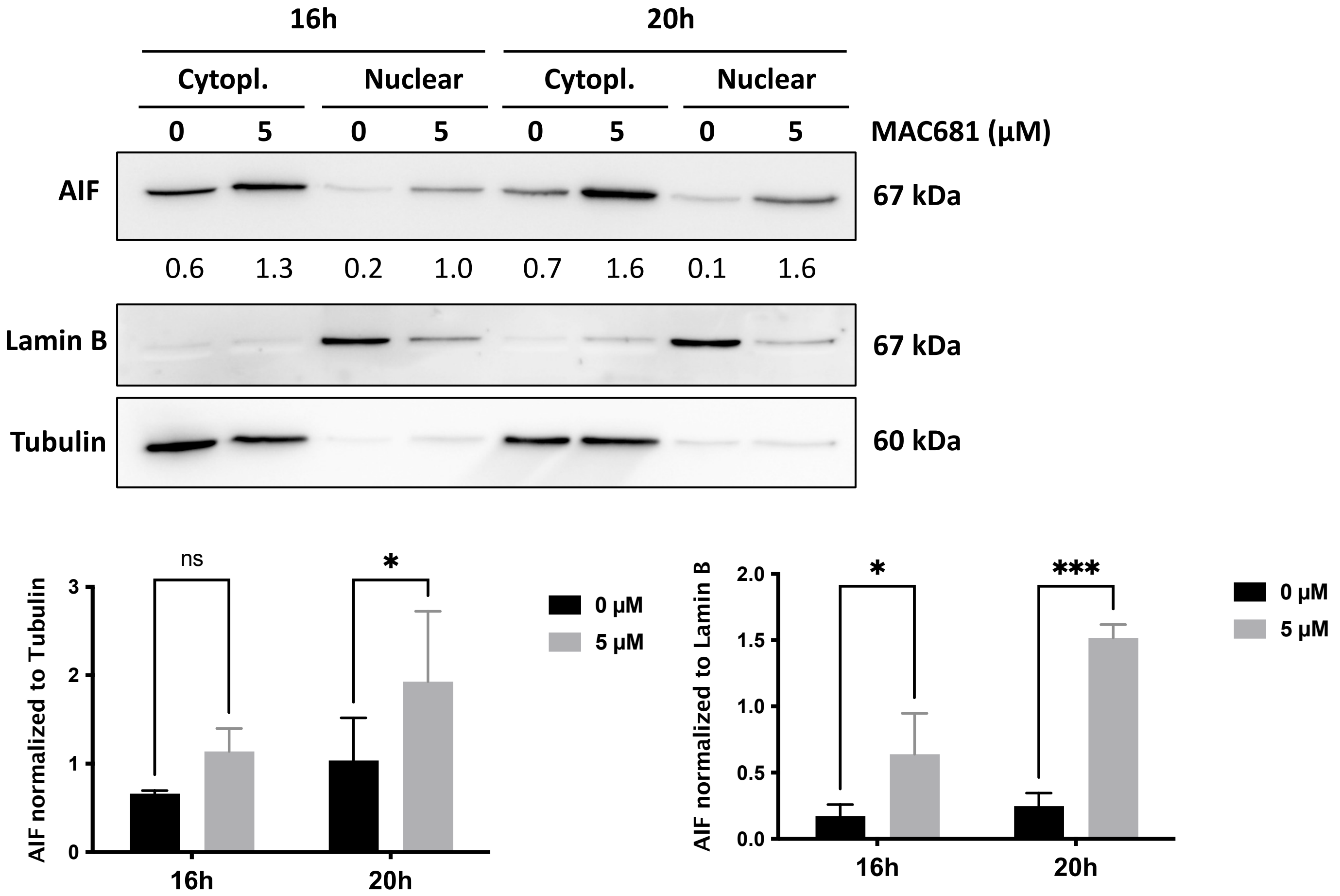

Supplementary figure

S2F

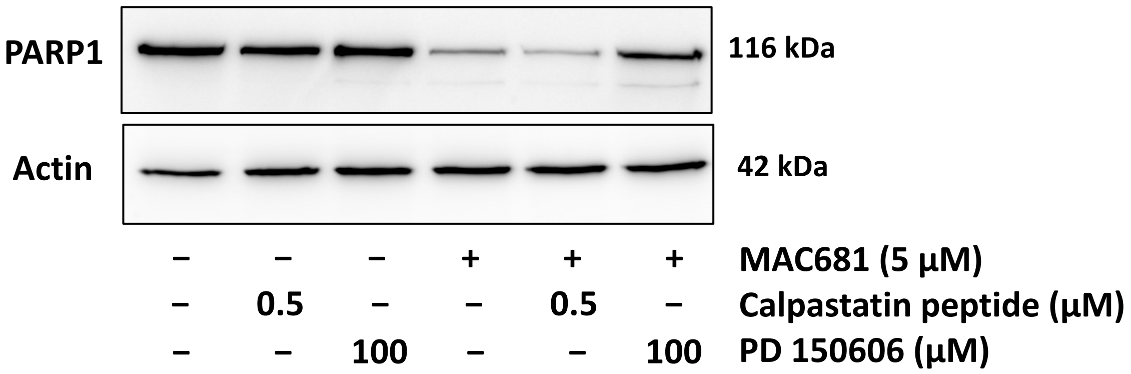

Supplementary figure

S2G

Cell lines	IC50 viability (µM)		
	**24h**	**48h**	**72h**
**K-562**	2.18 ± 0.17	1.25 ± 0.03	1.34 ± 0.04
**K-562R**	4.89 ± 0.40	2.28 ± 0.13	2.25 ± 0.10
**Jurkat**	1.95 ± 0.14	1.22 ± 0.04	1.30 ± 0.06
**U937**	5.92 ± 0.48	2.00 ± 0.11	1.57 ± 0.05

Cell lines	IC50 CTG (µM)		
	**24h**	**48h**	**72h**
**K-562**	1.29 ± 0.10	1.10 ± 0.09	1.49 ± 0.14
**K-562R**	2.54 ± 0.08	2.44 ± 0.18	4.03 ± 0.17
**Jurkat**	2.35 ± 0.20	1.13 ± 0.07	0.90 ± 0.04
**U937**	2.97 ± 0.18	1.17 ± 0.07	1.04 ± 0.05

S2H

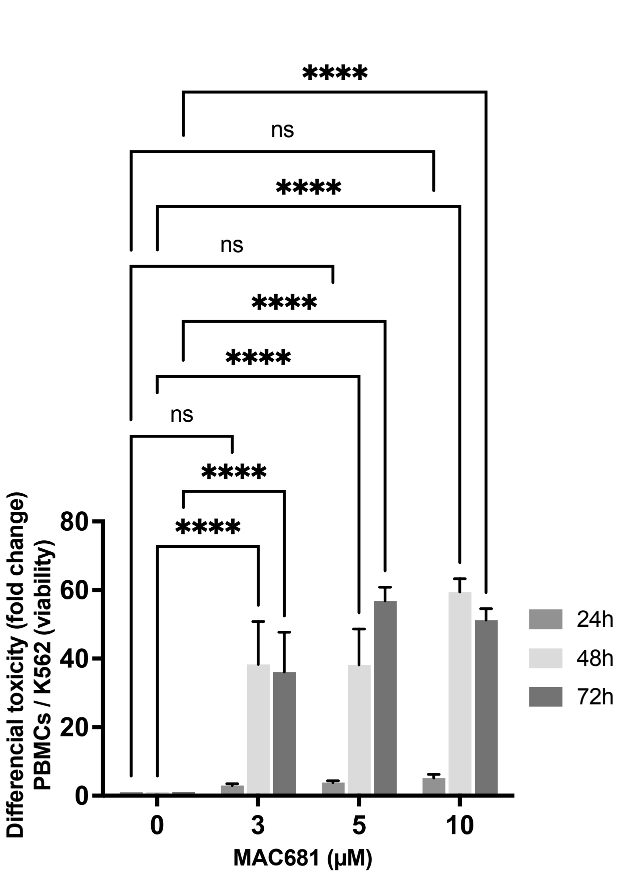

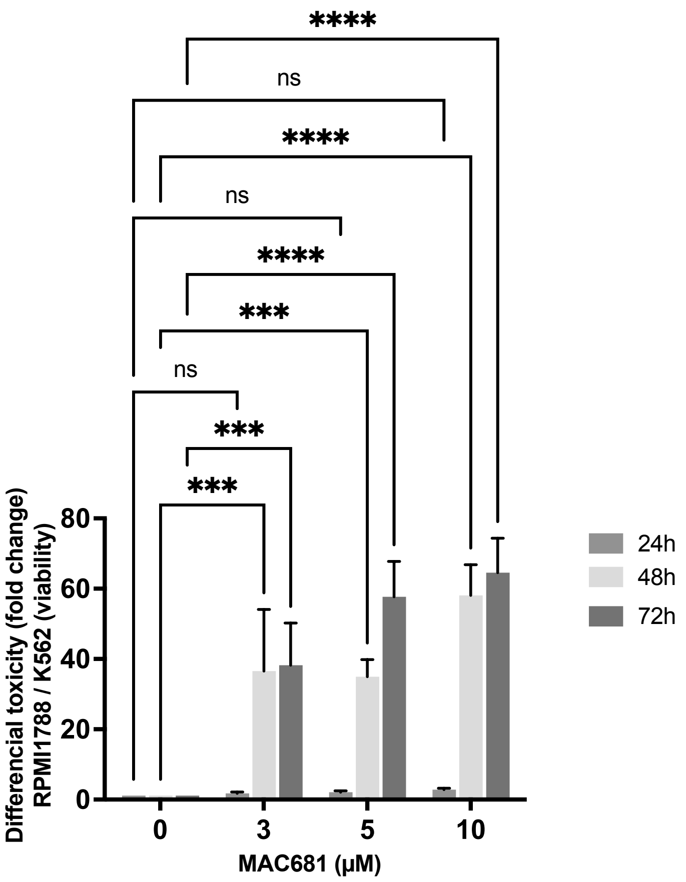

Supplementary figure

S2I

K-562

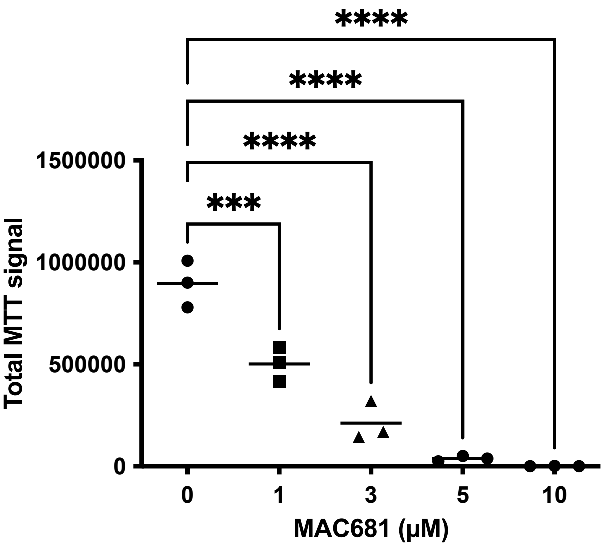

Supplementary figure

S2J

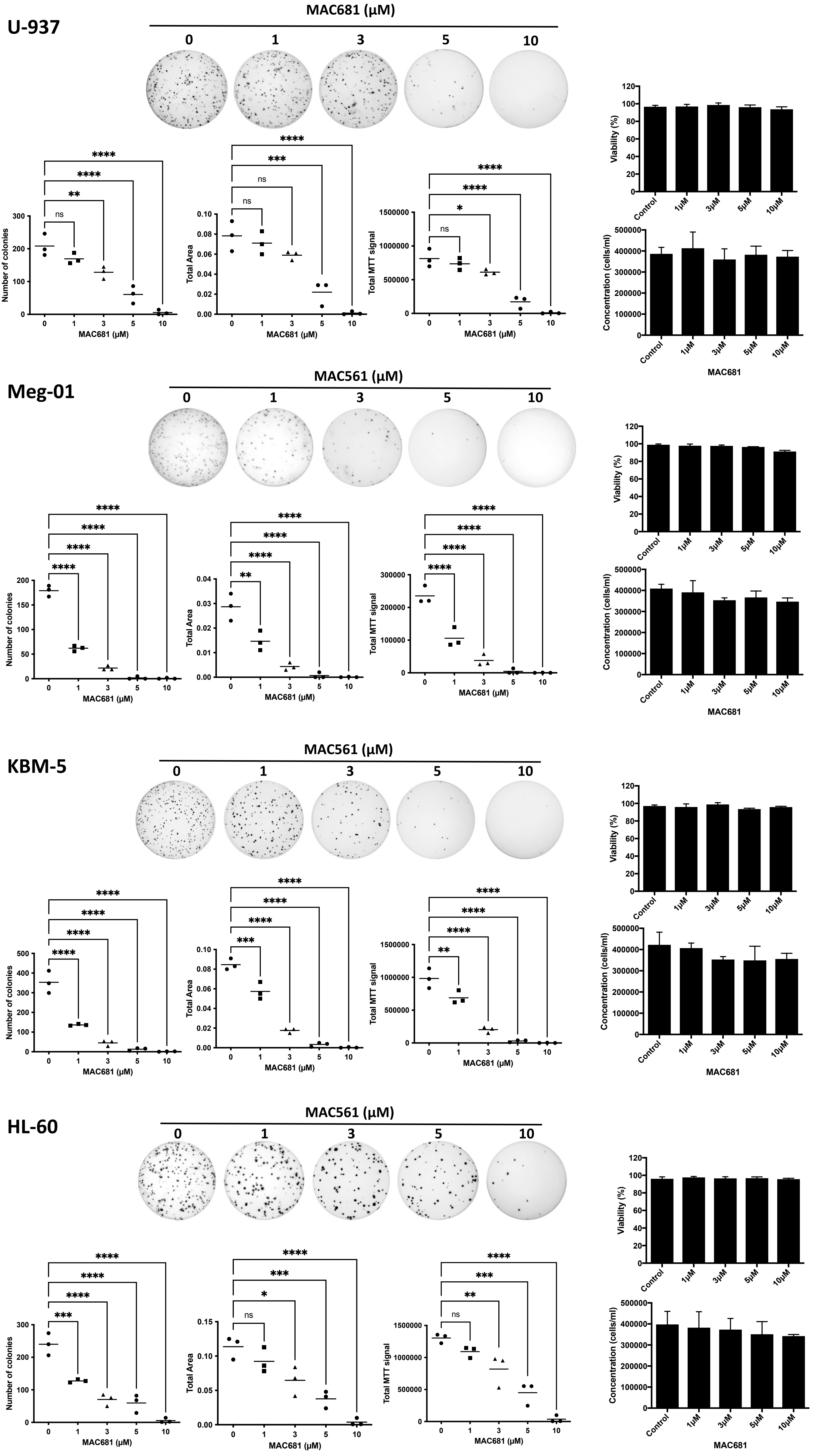

Supplementary figure

S2K

K-562

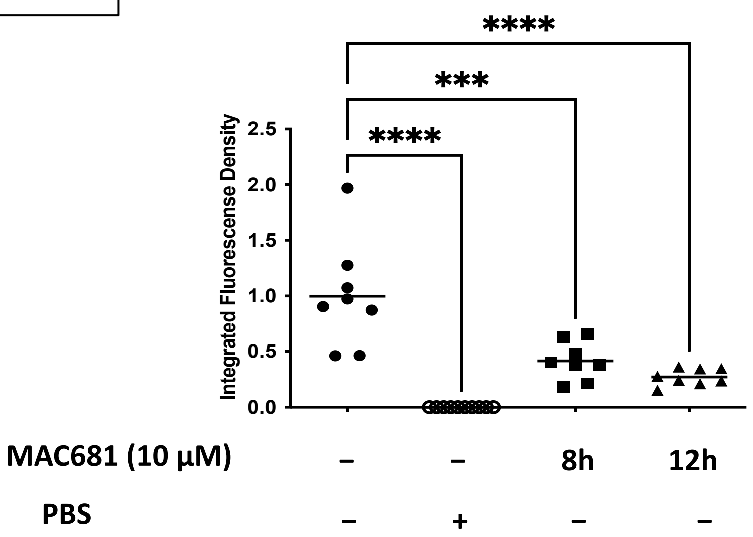

U-937

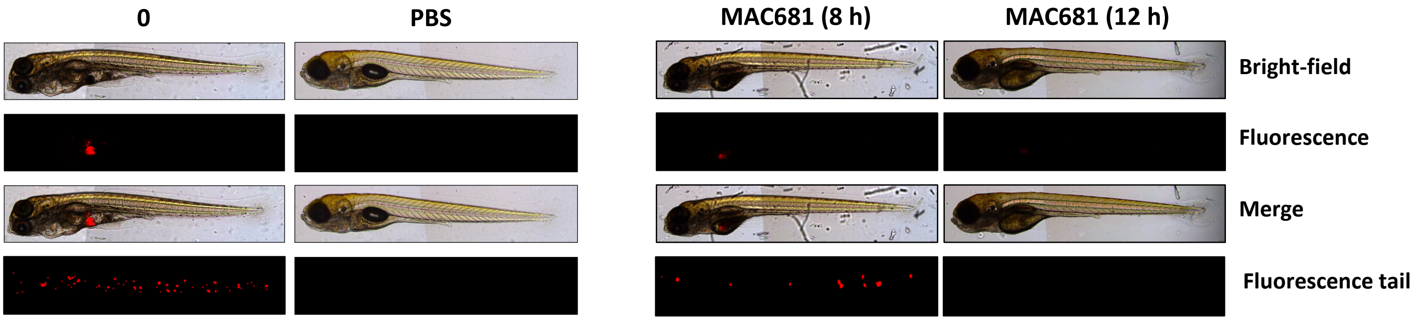

U-937-primary cells

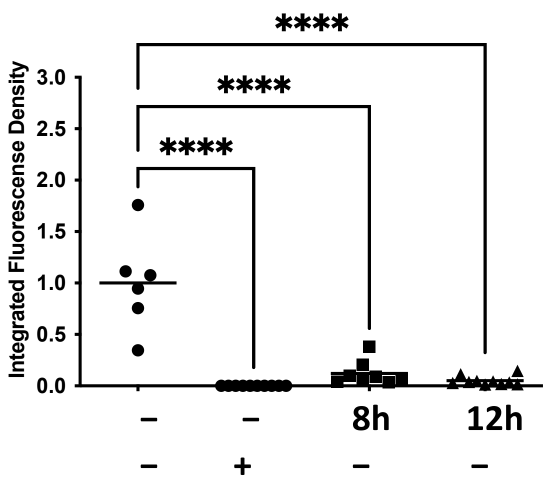

U-937-disseminated cells

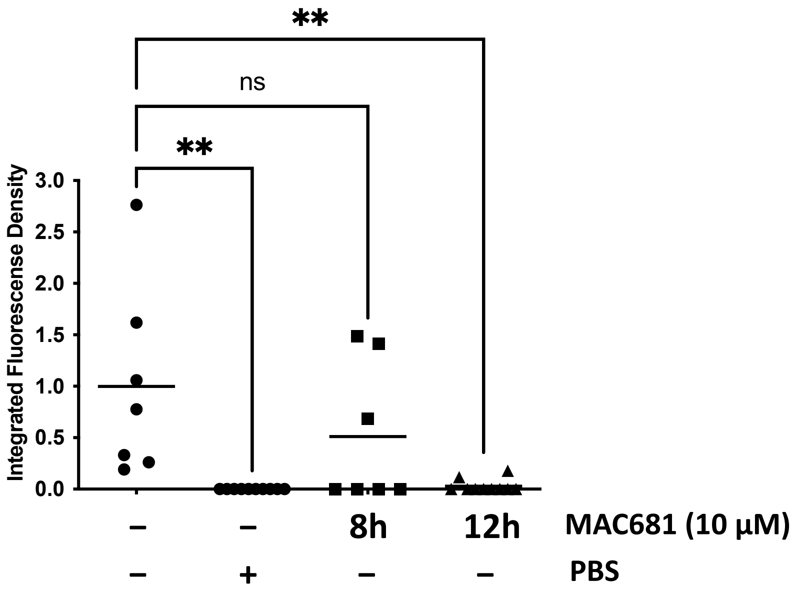

Supplementary figure
