# Supplementary material for: Antileukemic potential of methylated indolequinone MAC681 through immunogenic necroptosis and PARP1 degradation
Source: Biomark Res. 2024 May 4;12:47. doi: 10.1186/s40364-024-00594-w (PMC11069214; doi:10.1186/s40364-024-00594-w)
Supplement: Supplementary file 6 — Additional file 6. [file 40364_2024_594_MOESM6_ESM.docx]

**Supplementary information**

**Antileukemic potential of methylated indolequinone MAC681 through immunogenic necroptosis and PARP1 degradation**

Barbora Orlikova-Boyer^1^, Anne Lorant^1^, Sruthi Reddy Gajulapalli^2^, Claudia Cerella^1^, Michael Schnekenburger^1^, Jin-Young Lee^2+^, Ji Yeon Paik^2^, Yejin Lee^2^, David Siegel^3^, David Ross^3^, Byung Woo Han^2^, Nguyen Thi Kim Yen^2^, Christo Christov^4^, Hyoung Jin Kang^5^, Mario Dicato^1^, and Marc Diederich^2^

Table S1: Pharmacological inhibitors used in this study.

| Compound | Mode of action | Pre-treatment time | Stock concentration | Solvent | Reference |
| --- | --- | --- | --- | --- | --- |
| 3ABA | PARP1 inhibitor | 30 min | 100 mM | PBS | Calbiochem  165350 |
| zVAD | Pan caspase inhibitor | 1 h | 50 mM | DMSO | Calbiochem 627610 |
| VP16 | Apoptosis inducer | 24 h | 50 mM | DMSO | Sigma  E1383 |
| Cisplatin | chemotherapy drug | 24 h | 3.3 mM | PBS | Teva Pharmaceuticals  00703-5748-11 |
| Oxaliplatin | Immunogenic cell death inducer | 48h | 100 mM  (100 µM) | DMSO | Sigma  09512 |
| CCCP | OXPHOS uncoupler | 20 min | 10 mM | DMSO | Sigma  C2759 |

Working concentration are indicated in each experiment.

Table S2: Buffer solutions preparation for cytoplasmic and nuclear extraction.

| **Buffer solution A (total volume 10 ml)** | **Volume** |
| --- | --- |
| Hepes pH 7.9 (0.5M) | 200 μl |
| KCl (1M) | 100 μl |
| EDTA (0.5M) | 2 μl |
| EGTA (0.1M) | 10 μl |
| DTT (1M) | 10 μl |
| PMSF (0.2M) | 25 μl |
| Complete (Protease inhibitor cocktail) | 400 μl |
| ddH_2_O | 8.25 ml |
| PhosSTOP | 1ml (1 pill dissolved in 1ml ddH_2_O) |
| **Buffer solution C (total volume 1 ml)** | **Volume** |
| Hepes pH 7.9 (0.5M) | 40 μl |
| NaCl (1M) | 400 μl |
| EDTA (0.5M) | 2 μl |
| EGTA (0.1M) | 10 μl |
| DTT (1M) | 1 μl |
| PMSF (0.2M) | 5 μl |
| Complete (Protease inhibitor cocktail) | 40 μl |
| ddH_2_O | 202 μl |
| PhosSTOP | 100 μl (1 pill dissolved in 1ml ddH_2_O) |
| Glycerol | 200 μl |

Table S3: Antibodies used for western blots.

| **Antibody** | **Company** | **Catalog number** | **Dilution** |
| --- | --- | --- | --- |
| PARP1 | Cell Signaling | 9542S | 1:1000 in 5% milk |
| PARP1 (C2-10) | Santa Cruz biotechnology | sc-53643 | 1:1000 in 5% milk |
| Mcl-1 | Cell signaling | 4572 | 1:1000 in BSA |
| Bcl-2 | Calbiochem | OP60 | 1:2000 in milk |
| Bcl-xL | BD Biosciences | 610212 | 1:1000 in milk |
| AIF (E-1) | Santa Cruz biotechnology | sc-13116 | 1:1000 in milk |
| β-actin | Sigma | A5441 | 1:10000 in 5% milk |
| Lamin B  (C-20) | Santa Cruz biotechnology | sc-6216 | 1:1000 in milk |
| α-Tubulin  (DM1A) | Calbiochem | CP06 | 1:5000 PBS-T |
